# Human Food Safety Implications of Variation in Food Animal Drug Metabolism

**DOI:** 10.1038/srep27907

**Published:** 2016-06-15

**Authors:** Zhoumeng Lin, Christopher I. Vahl, Jim E. Riviere

**Affiliations:** 1 Institute of Computational Comparative Medicine (ICCM), Department of Anatomy and Physiology, College of Veterinary Medicine, Kansas State University, Manhattan, KS 66506, USA; 2 Department of Statistics, College of Arts and Sciences, Kansas State University, Manhattan, KS 66506, USA

## Abstract

Violative drug residues in animal-derived foods are a global food safety concern. The use of a fixed main metabolite to parent drug (M/D) ratio determined in healthy animals to establish drug tolerances and withdrawal times in diseased animals results in frequent residue violations in food-producing animals. We created a general physiologically based pharmacokinetic model for representative drugs (ceftiofur, enrofloxacin, flunixin, and sulfamethazine) in cattle and swine based on extensive published literature. Simulation results showed that the M/D ratio was not a fixed value, but a time-dependent range. Disease changed M/D ratios substantially and extended withdrawal times; these effects exhibited drug- and species-specificity. These results challenge the interpretation of violative residues based on the use of the M/D ratio to establish tolerances for metabolized drugs.

The cornerstone of regulatory chemical food safety programs is the monitoring of food products for violative chemical residues. For edible products from food-producing animals, such as meat, milk or eggs, residue concentrations are determined based on jurisdictional-specific regulations that result in the determination of a tolerance (TOL) or maximum residue level (MRL) for specific drugs in a specific tissue for specific animal species^1^. These are based on toxicological assessments related to consumption of meat containing the specific drug in the diet coupled to a safety factor that accounts for uncertainty which yields the acceptable daily intake (ADI). These calculations using laboratory animal dietary toxicity studies to determine no observed adverse effect levels are based on exposure to total drug, which is the sum of parent drug and metabolites. From this total ADI, food consumption is used to calculate what the TOL or MRL for total drug exposure in so-called target tissues (e.g., liver, kidney, muscle). When the drug under question is metabolized in the food animal species, a ratio is established very early in the drug development process between total drug and the “marker residue”, which would be monitored for analytical determination in tissue testing. The marker residue could be either the parent drug or a metabolite. The legal TOL and MRL are then expressed in terms of this marker residue.

There are a number of potential issues surrounding the use of a marker residue and determination of the main metabolite to parent drug (M/D) ratio (a surrogate of the ratio of the marker residues to total residues). These include: (i) the ratio is determined in a very early study for limited early time points in a small number of animals and usually assumed as a fixed parameter, (ii) the studies are done in healthy animals, but the drug is used in diseased animals, and (iii) the final formulation is not employed. Subsequent studies are then conducted employing this fixed ratio in larger number of healthy animals to determine a withdrawal time after cessation of drug administration that will deplete target tissues to below the TOL or MRL for the marker residue. Due to these flaws, violative drug residues in the edible tissues of food-producing animals may occur when drugs are used in an extralabel manner or when disease is present even though the animals are slaughtered according to regulatory labeled withdrawal times^2^,^3^. Commonly reported drug violations include ceftiofur, enrofloxacin, flunixin, and sulfamethazine^4^. As a result, drug residue violation in food animals has become a global food safety concern^5^,^6^.

The focus of this study is to provide systematic evidence that the use of a fixed M/D ratio in the calculation of TOL (or MRL) and withdrawal times is fundamentally flawed. We hypothesized that changes in the rate and extent of drug metabolism due to disease or species (or even breed) differences could alter this ratio, tissue residue levels and withdrawal times, making the marker residue a poor indicator of tissue exposure to unsafe drug concentrations.

## Results

### Calibration of a general physiologically based pharmacokinetic (PBPK) for multiple drugs in two food animal species

**Figure 1:**
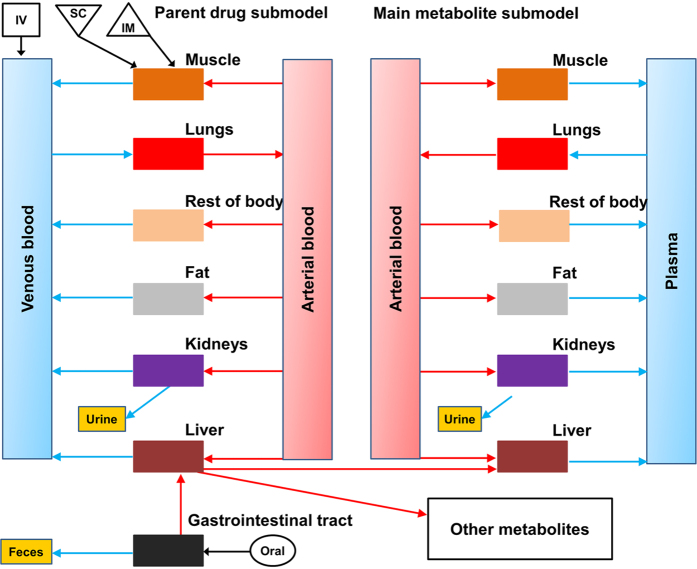
A schematic of a general physiologically based pharmacokinetic (PBPK) model for drugs approved in food animals that have more than one metabolite in the body. PBPK models for several representative drugs, including ceftiofur, enrofloxacin, flunixin and sulfamethazine, in cattle and swine were developed and evaluated in this study. IV, IM, SC, and oral represent drug administration through intravenous, intramuscular, subcutaneous injection, and oral route (gavage or feed), respectively.

**Figure 2:**
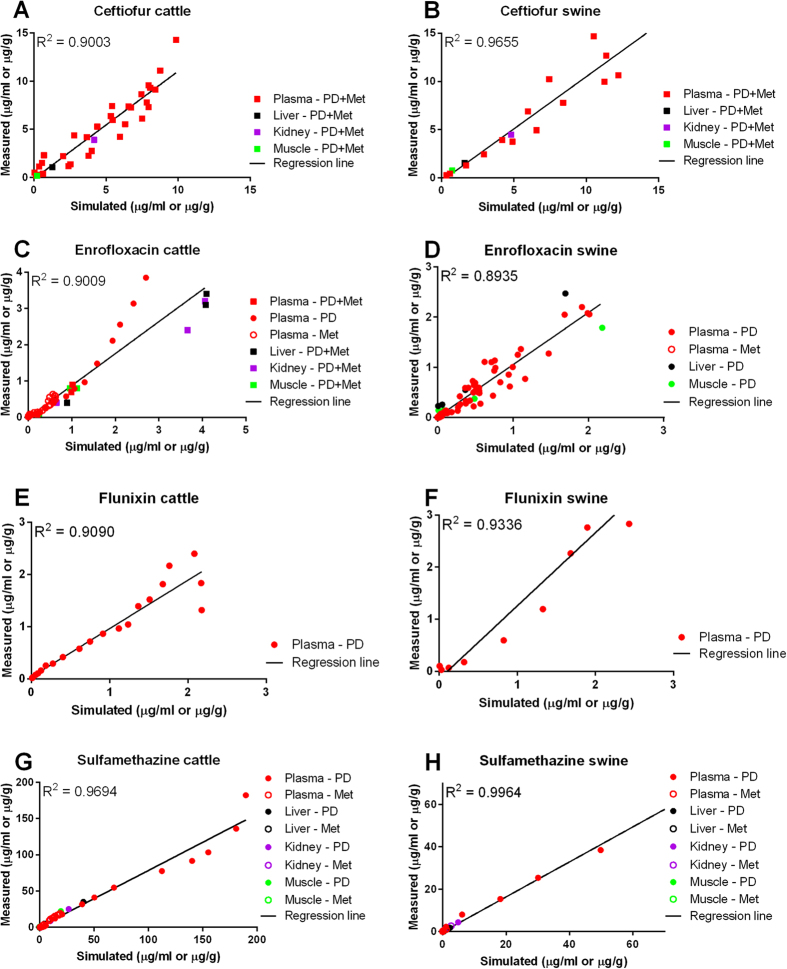
Regression analysis results of PBPK model calibration datasets. PBPK models for ceftiofur (**A**,**B**), enrofloxacin (**C**,**D**), flunixin (**E**,**F**), and sulfamethazine (**G,H**) in healthy cattle and swine were developed based on datasets listed in Supplementary Table 1. Each panel represents the result of a regression analysis between model-simulated and measured plasma and/or tissue drug concentrations for each drug in each species. R^2^ values and regression lines are shown in each panel. PD: concentrations of the parent drug; Met: concentrations of the main metabolite; PD+Met: concentrations of parent drug plus its major metabolite.

To predict the M/D ratio and evaluate how possible change of this ratio by disease is related to drug withdrawal times, we established a general PBPK computational model for several commonly reported residue-violative drugs (ceftiofur, enrofloxacin, flunixin, and sulfamethazine) in cattle and swine (Fig. 1). The model was calibrated for each chemical in each species with multiple datasets from the Food Animal Residue Avoidance Databank (FARAD) (listed in Supplementary Table 1). All model parameters are provided in Supplementary Tables 2–6 and details of model development refers to Methods. Results of regression analyses between model-simulated and measured plasma and tissue concentrations of parent drugs and/or major metabolites for each drug in each species are shown in Fig. 2. All determination coefficient R^2^ values were ≥0.89, indicating excellent overall goodness-of-fit for all simulations. Comparisons of the time course of model-simulated versus measured concentration data are shown in Supplementary Fig. 1 (ceftiofur in cattle), Supplementary Fig. 2 (ceftiofur in swine), Supplementary Fig. 3 (enrofloxacin in cattle), Supplementary Fig. 4 (enrofloxacin in swine), Supplementary Fig. 5 (flunixin in cattle), Supplementary Fig. 6 (flunixin in swine), Supplementary Fig. 7 (sulfamethazine in cattle), and Supplementary Fig. 8 (sulfamethazine in swine). Overall, the model-simulated concentrations correlated with the measured data very well for all selected drugs in both species.

### Evaluation of the PBPK model with independent data

The model was further employed to simulate other pharmacokinetic studies not used in the model calibration process (Supplementary Table 1). As shown in Supplementary Fig. 9, all R^2^ values for the regression analyses between model-predicted and measured data were ≥0.76, suggesting adequate goodness-of-fit. These model evaluation results suggest that our model can be used to predict the concentrations of studied drugs and/or their metabolites in the plasma and tissues of cattle and swine with an acceptable accuracy. Model sensitivity analyses were also performed and the results are presented in Supplementary Table 7.

### PBPK model-predicted M/D ratios of studied drugs exhibit a wide range, depend on multiple factors, and correlate with experimental data well

After model validation, we applied the model to predict the M/D ratios of studied drugs and compared the predicted results to measured data (reported M/D ratio data for studied drugs are shown in Supplementary Table 8). The results showed that simulated M/D ratios for enrofloxacin, flunixin, and sulfamethazine had a wide range and were dependent on the species, drug, exposure route, tissue, and time, which correlated with experimental data very well (Supplementary Fig. 10). For example, the M/D ratios for enrofloxacin in the plasma of cattle after IV injection (5 mg/kg) was ~0.1–10, but these ratios were generally <0.2 in the plasma of swine exposed to the same dose of enrofloxacin via the same route. Within the same species, the M/D ratios were very close in the plasma of swine exposed to 5 mg/kg enrofloxacin via IV versus to 10 mg/kg orally, but the M/D ratios were about 2-fold different between subcutaneous (SC) and IV exposures to the same dose of flunixin (2.2 mg/kg). The M/D ratios for sulfamethazine kept changing during the absorption phase (≤168 h) and the distribution phase (168–200 h), but it seemed to be constant during the terminal phase (≥200 h) in the plasma or tissues of swine after a 7-day consecutive oral exposure at 12-h intervals (Supplementary Fig. 10). In addition, the M/D ratios in the kidney were ~5-fold higher than in the muscle of swine after 7-day oral exposure to sulfamethazine.

### Disease changes the M/D ratio substantially and the degree of changes depends on the drug, exposure time, tissue and species

**Figure 3:**
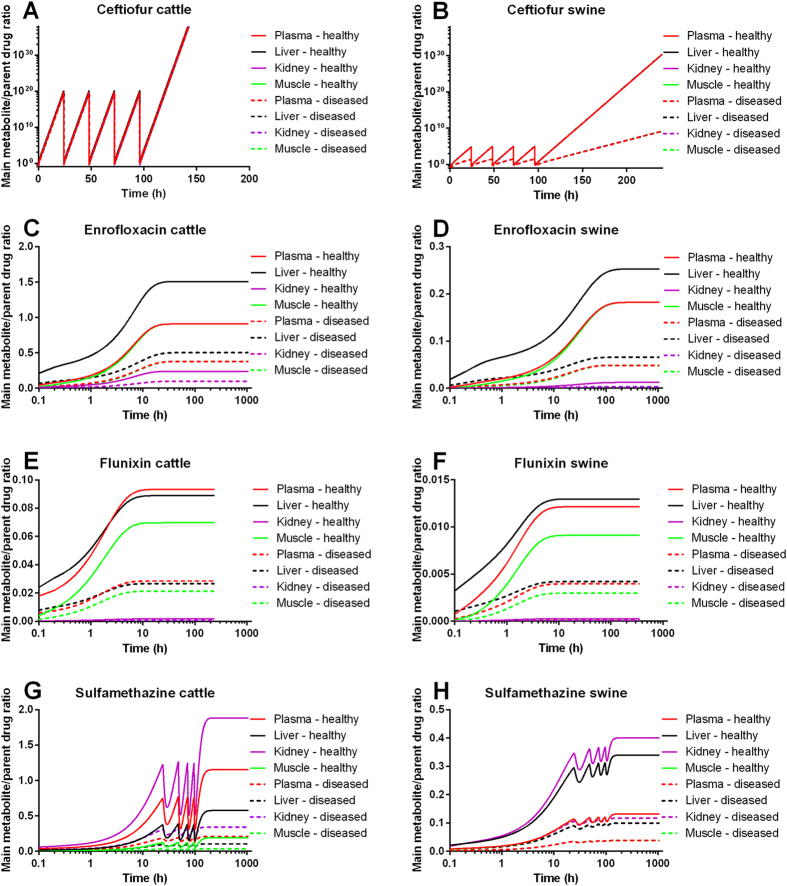
Effect of disease on the main metabolite to parent drug (M/D) ratio. Healthy and diseased cattle and swine were assumed to be exposed to labeled therapeutic regimens of ceftiofur (**A,B**), enrofloxacin (**C,D**), flunixin (**E,F**), and sulfamethazine (**G,H**). The M/D ratios of studied drugs in the plasma and tissues of cattle and swine were predicted using the PBPK model. The labeled therapeutic regimens of these drugs are shown in Supplementary Table 9.

Next, we employed the model to predict the M/D ratio and tissue residues of studied drugs in the plasma and tissues in each species in both healthy and diseased animals after exposure to representative labeled therapeutic paradigms. Information about labeled therapeutic regimens, marker residues, target tissues, withdrawal times, and TOL of studied drugs is provided in Supplementary Table 9. The results showed that disease altered the M/D ratio substantially, regardless of the drug, administration regimen, exposure time, species, and tissue (Fig. 3 and Table 1). The degree of alterations varied depending on the drug, exposure time, tissue, and species. In general, disease changed the M/D ratios of ceftiofur, enrofloxacin, flunixin, and sulfamethazine by several (i.e., 2–6) fold and the M/D ratio could be different by up to several orders of magnitude at the withdrawal time compared to that at the time right after drug administration (the first simulation time was at 0.1 h after drug administration). For example, diseased changed the plasma M/D ratio of ceftiofur in cattle by 2-fold and in swine by 3-fold at 0.1 h after drug administration, and diseased altered the plasma M/D ratio of sulfamethazine by ~5.5-fold in cattle and by ~3-fold in swine at the withdrawal time. Note that for ceftiofur the model-predicted M/D ratios at the withdrawal time could not be accurately estimated (i.e., >10^20^) because ceftiofur’s metabolic transformation was so rapid that at the withdrawal time, the residue was essentially all metabolites (Table 1).

### Disease affects the tissue residue levels and estimated withdrawal times in a drug- and species-dependent manner

**Figure 4:**
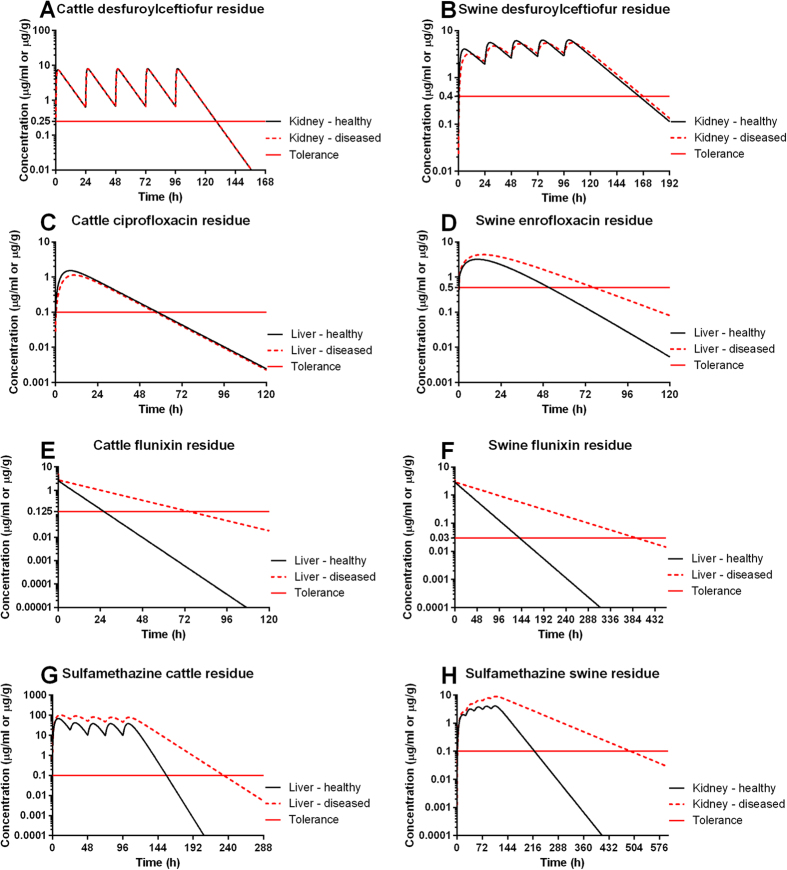
Effect of disease on the tissue residue and withdrawal time. Healthy and diseased cattle and swine were assumed to be exposed to labeled therapeutic regimens of ceftiofur (**A,B**), enrofloxacin (**C,D**), flunixin (**E,F**), and sulfamethazine (**G,H**). The concentrations of marker residues of studied drugs in the target tissue were predicted using the PBPK model. The predicted marker residue concentrations were compared to the tolerances (red solid lines) of studied drugs in each species. The labeled therapeutic regimens of studied drugs are shown in Supplementary Table 9.

The PBPK model-simulated results also showed that disease affected the drug tissue marker residue levels, which in turn impacted the withdrawal time estimation, but this effect was dependent on the drug and species (Fig. 4). Specifically, disease did not (or minimally) affect the concentration of ceftiofur marker residue (its main metabolite desfuroylceftiofur) in the cattle and swine. Disease did not change the concentration of the marker residue of enrofloxacin (its main metabolite ciprofloxacin) in cattle, either, but the enrofloxacin marker residue in swine is the parent drug and its concentrations were considerably altered by disease. As a result, the withdrawal time for enrofloxacin in diseased swine should be extended by at least 1 day (Fig. 4). The marker residue for flunixin and sulfamethazine in both species is the parent drug itself. Disease substantially changed the marker residues of flunixin and sulfamethazine in the target tissues of both cattle and swine. Consequently, based on the data used in these simulations, the withdrawal time should be prolonged by 3 days for flunixin in cattle, by 11 days for flunixin in swine, by 4 days for sulfamethazine in cattle, and by 12 days for sulfamethazine in swine (Fig. 4). These results suggest that the withdrawal time can be substantially lengthened due to disease for certain drugs (e.g., flunixin and sulfamethazine) with the parent compound as the marker residue. Therefore, it is scientifically flawed to label withdrawal times determined in healthy animals for use in diseased animals.

## Discussion

The model simulations suggest that the M/D ratio is not a fixed value, but has a wide range, depending on multiple factors, including the drug, exposure time, species, and disease state. This finding is important because it challenges the fundamental assumption of using a fixed point estimate for the M/D ratio used in the current US FDA guideline for evaluating the safety of compounds in food-producing animals^1^. According to this guideline, a marker residue is a residue whose concentration is in a known relationship to the concentration of the total residue in the last tissue to deplete to its permitted concentration^1^. The marker residue can be the parent drug, any of its metabolites (usually the main metabolite), or a combination of the residues. Our data clearly show that this relationship is not a fixed point estimate, but rather varies over a wide range because of the numerous factors influencing it. In particular, our data demonstrate that the M/D ratio can be different by several fold to several orders of magnitude at the withdrawal time compared to that at the time right after drug administration and disease and route of administration can change the M/D ratio by several fold in both plasma and tissues regardless of the drug or species. Thus, the withdrawal time calculated based on a fixed M/D ratio in healthy animals is not appropriate for diseased animals.

It should be noted that the drawback of using a ratio involving metabolites to determine a withdrawal/detection time has already been challenged in other fields, such as doping control in horses^7^. The influence of diseases on the disposition of veterinary drugs and the resulting consequences on withdrawal time has been acknowledged for several decades^8^. The strength of the present study is to provide a broader-based quantitative estimate of the magnitude of this effect based on PBPK model simulations involving multiple veterinary drugs in two commonly used food animal species. Overall, our results strongly suggest that the current FDA guideline on the withdrawal time determination^1^ may need to be revised.

The fact that the M/D ratio is altered substantially due to disease is mainly because disease changes the hepatic metabolism of drugs. The extent of changes in the M/D ratio depends on the severity of the disease. The present study assumed an average 3-fold change in hepatic metabolic rate based on the available published pharmacokinetic studies in diseased animals (further justified in Methods)^2^,^9^,^10^. It should be noted that in reality some animals may have very severe diseases and the extent of changes in hepatic metabolism may be greater, which, in turn, may alter the M/D ratio and withdrawal time even more. Additionally, hepatic metabolism of studied drugs was described using a linear equation because their metabolism is generally not saturated at therapeutic doses (justified in Methods). However, if saturation occurs, the M/D ratios might be even skewed more.

Besides diseases, other factors, such as genetic polymorphisms, breed differences, the type of food, drug-drug interaction, and feedlot environment, may also affect M/D ratios and withdrawal times^11^,^12^,^13^,^14^. Recent studies have proposed population-based approaches, either population mixed-effect pharmacokinetic^15^,^16^ or population PBPK modeling^17^,^18^, to estimate withdrawal times of veterinary drugs in food animals because population-based approaches are stochastic modeling techniques that can consider all possible factors and incorporate all available data into analysis. Population-based analysis of the present PBPK models was not performed because the focus on this work was to evaluate the M/D ratio variation and how it was affected by disease. Nevertheless, the present model provides a foundation for population-based analysis, which is a future objective.

Another merit of this study is the creation of a general multi-route PBPK model for multiple drugs from different use classes, including cephalosporins (ceftiofur), fluoroquinolones (enrofloxacin), nonsteroidal anti-inflammatory drugs (flunixin), and sulfonamides (sulfamethazine) across two common food animal species. This modeling strategy represents a significant advance in this field because published PBPK models for veterinary drugs all focus on a single drug or one species^18^,^19^,^20^,^21^,^22^,^23^. These available models have different model structures that prevent direct comparisons of the pharmacokinetics of different drugs across species. It is also time-consuming, expensive, and may be unrealistic to develop individual models for each drug because there are numerous drugs in the market. In this regard, the use of a general model is crucial because it allows to comparing pharmacokinetic parameters of various drugs between species. A general model also makes it economically affordable and technically more feasible to assess M/D ratios and withdrawal times of multiple drugs using PBPK modeling approach. Successful validation of the present model in two food animal species suggest that this model can also be extrapolated to other food animal species, such as goat, sheep, and poultry. Therefore, the present model represents a novel and useful tool in veterinary pharmacometrics and will increase the application of PBPK modeling in veterinary medicine and food safety.

As discussed above, our simulation data also bring into question the toxicological interpretation of violative tissue residues in food safety surveillance programs determined using TOL or MRL of drugs known to be metabolized in food animals. The determination of actual risk to human consumption of these violative residues is calculated based on total drug, and must be interpreted in the context of flawed assumption of a fixed M/D ratio, which could suggest violation of the marker residue, despite safe total drug concentrations and thus no real risk to the consumer. This also illustrates the complexity facing regulators for determining TOL and/or withdrawal times for heavily metabolized pesticides in food-producing animals, which are currently not available and need to be established. The present study provides a framework for extrapolating to environmental contaminants (i.e., pesticides), other drugs, and to other species.

In summary, the present study demonstrated that the M/D ratio of studied drugs (ceftiofur, enrofloxacin, flunixin, and sulfamethazine) was not a fixed value, but rather varied over a wide range and was dependent on numerous factors, including disease, species, drug, tissue, exposure route, and time using a newly established general PBPK model. These results raise a question about the reasonableness of the underlying assumption of using a fixed M/D ratio parameter by FDA to determine TOL and withdrawal times of veterinary drugs in food animals. Nevertheless, it should be acknowledged that although the FDA method for calculating withdrawal times assumes that the M/D ratio is fixed and requires residue depletion studies be conducted in healthy animals, it is a relatively conservative estimate because the tolerance limit requires that 99% of the treated population will have residues below the tolerance at the labeled withdrawal time with a 95% confidence^1^. Further studies that utilize the present model framework and the proposed population-based modeling techniques are warranted to provide more reasonable estimates of drug withdrawal times that reflect actual field conditions of drug use to aid regulatory decisions on food safety.

## Methods

### Experimental data source

Pharmacokinetic data for ceftiofur, enrofloxacin, flunixin, and sulfamethazine in healthy and diseased cattle and swine after intravenous (IV), intramuscular (IM), subcutaneous (SC), or oral exposure were obtained from the Food Animal Residue Avoidance Databank (FARAD) comparative pharmacokinetic database, a USDA-supported initiative in veterinary medicine^24^,^25^. Key information of selected studies is provided in Supplementary Table 1. All data were extracted from selected studies using WebPlotDigitizer (version 3.8, http://arohatgi.info/WebPlotDigitizer/). Only data from the heathy animals and values above the limit of quantification (generally LOQ ≥ 0.1 μg/ml) were used for model calibration and evaluation.

### Model structure

The model structure for ceftiofur, enrofloxacin, flunixin and sulfamethazine was designed to include two sub-models for the parent drug and the major metabolite, respectively, with each sub-model consisting of 7 compartments, including the plasma, liver, kidney, muscle, fat, lung, and rest of body (Fig. 1). The other minor metabolites were pooled together and modeled as a single compartment. The liver, kidney, muscle and fat were modeled as individual compartments because these organs are common edible tissues and relevant to food safety. Plasma was included as it is the essential compartment linking all other compartments through systemic circulation. The lung was considered as one compartment because many drugs are used to treat pneumonic infections, for which the lung is the target organ of interest. Additionally, it was necessary to include a lumped compartment to account for disposition of the drug and its metabolite to the rest of body. All compartments were assumed to be blood flow-limited and well-stirred.

In order to develop a general PBPK model, common drug administration routes, including IV, IM, SC, and oral (gavage or via feed) exposures were included in the model. Note that besides therapeutic administrations via IV, IM, SC and oral routes, other causes of drug tissue residues include management practices, feed supplies, and recycling through bedding, which are important confounding factors for the establishment of a withdrawal time in doping control and is relevant to drug tissue residue violation (especially for drugs extensively eliminated by renal clearance)^11^. However, the contribution of drug concentrations from these other causes are almost negligible compared to that from therapeutic administrations. Therefore, these other possible exposure sources, such as through recycling of bedding, were not included in the present PBPK model.

Plasma protein binding of parent drugs and their main metabolites was simulated based on experimentally measured fixed ratio values^20^,^26^,^27^. Elimination pathways included hepatic metabolism of parent drugs, urinary excretion of parent drugs and their metabolites via the kidney. Since selected drugs have multiple metabolites, a percentage parameter (i.e., Frac) was included as an initial estimate to represent the fraction of a parent drug that is metabolized to form its major metabolite. The metabolic and excretory processes were described using first-order linear equations^22^,^28^. The therapeutic dose window of veterinary drugs is typically narrow and their metabolism is generally not saturated at therapeutic doses, so a linear equation is often sufficient and commonly used to simulate metabolism of veterinary drugs in food animals^18^,^22^,^23^. The model was implemented using acslX software (Version 3.0.2.1, Aegis Technologies Group, Inc., Huntsville, AL). Equations and complete model code describing the absorption, plasma protein binding, distribution, metabolism, and elimination processes are provided and explained in the Supplementary Information. The complete model code will also be deposited on our website (http://iccm.k-state.edu/).

### Model parameterization

Physiological parameters for cattle and swine were obtained from the literature^18^,^20^,^22^ and are provided in Supplementary Table 2. Initial values for partition coefficients were obtained from earlier PBPK models for flunixin and sulfamethazine^20^,^22^, or calculated using tissue:plasma AUC (area under the concentration curve) ratio method based on pharmacokinetic data in cattle for enrofloxacin^29^, or based on the reported tissue penetration factors for ceftiofur^27^. Initial values for partition coefficients of main metabolites were not available and thus were assumed to be the same or similar to their parent drugs. Partition coefficients and other drug-specific parameters (route-specific absorption rates, hepatic metabolic rates, fraction of drugs metabolized to main metabolites, and elimination rates) were then estimated using the parameter optimization module in acslX, followed by an iterative manual adjustment approach to obtain a visually reasonable fit to the experimental data for model calibration. The chemical-specific parameter values are given in Supplementary Tables 3–6.

### Model evaluation

The goodness-of-fit of model predictions was evaluated by model convergence, visual inspection (i.e., whether the simulation result captures the kinetic profile of the experimental data), and determination coefficients (R^2^ values) of linear regression analyses between model-predicted and experimental data using GraphPad Prism 6 (GraphPad Software Inc., La Jolla, CA)^23^. Importantly, multiple independent datasets were used to evaluate model prediction performance for each drug in each species (listed in Supplementary Table 1). The criterion of a validated model was based on World Health Organization (WHO) PBPK modeling guidelines, i.e., if the simulated values were generally within a factor of two of the measured data, the model was considered acceptable and validated^30^.

### Sensitivity analysis

Sensitivity analysis was conducted to determine the effect of each parameter on critical model outputs, including 24-h AUC of plasma, liver, kidney, and muscle concentrations of parent drugs and main metabolites. The normalized sensitivity coefficient (NSC) was calculated using the following equation: NSC = Δr/r * p/Δp, where p is the initial parameter value, Δp is 1% of the parameter value, r is the model output derived from the original parameter value, and Δr is the change of model output due to 1% increase in the parameter value. Parameters with absolute values of NSC ≥0.5 were considered highly sensitive^28^.

### Model application

To evaluate whether the M/D ratio is a fixed value, the models were employed to predict the M/D ratio for ceftiofur, enrofloxacin, flunixin and sulfamethazine in the plasma and/or tissues of cattle and swine. The M/D ratio was used as a surrogate for the ratio of the marker residues to the total drug residues (parent drug plus all metabolites). The predicted values were compared to experimental data shown in Supplementary Table 8. Measured M/D ratio for ceftiofur is not available because most pharmacokinetic studies of ceftiofur measure the concentration of total desfuroylceftiofur acetamide as a composite of free desfuroylceftiofur, desfuroylceftiofur cysteine conjugate, plasma protein-bound desfuroylceftiofur, and a small fraction of other polar metabolites. There are no data measuring levels of both ceftiofur and its individual metabolites. Nevertheless, PBPK modeling made it possible to predict the M/D ratio for ceftiofur.

The present model can also be used to assess the effect of changes in the rate and extent of metabolism due to disease, genetic polymorphism, and/or breed/sex differences on the M/D ratios, tissue residue levels, and withdrawal times of studied drugs. Numerous studies have shown that disease alters the pharmacokinetics of various drugs^2^,^10^,^15^,^31^. The degree of pharmacokinetic changes depends on the severity of the disease. For example, chemical-induced acute hepatic or renal failure resulted in increased AUC and decreased clearance by ~2.2-fold in rats after IV administration of flunixin^32^. Nephrectomized rats had elevated AUC and reduced clearance by ~40% after enrofloxacin IV injection^33^. Mastitic cows had ~2-fold increase in AUC and ~2-fold decrease in clearance compared to healthy cows after IV flunixin administration^2^. Overall, a ~2-fold pharmacokinetic alteration was observed in diseased animals, and this change has been attributed to downregulation of hepatic and intestinal drug-metabolizing enzymes, such as ~40–85% decrease of total CYP450 activity^34^,^35^. Through iterative model simulations, it was found that a 3-fold decrease in the hepatic metabolism rate (Km [h^−1^]) was needed in order to achieve a 2-fold increase in model-predicted plasma AUC of the parent drug. Therefore, the model was used to assess the impact of 3-fold change of Km (designated as a general metabolic feature for diseased animals compared to healthy animals) on the M/D ratios, tissue residues, and withdrawal times of studied drugs in both cattle and swine according to the labeled therapeutic regimens (Supplementary Table 9).

## Additional Information

**How to cite this article**: Lin, Z. *et al*. Human Food Safety Implications of Variation in Food Animal Drug Metabolism. *Sci. Rep.* **6**, 27907; doi: 10.1038/srep27907 (2016).

## Supplementary Material

Supplementary Information

## Figures and Tables

**Table 1:** Main metabolite to parent drug (M/D) ratios of ceftiofur, enrofloxacin, flunixin, and sulfamethazine in healthy and diseased cattle and swine.

Drug	Species	Status	Plasma		Liver		Kidney		Muscle	
			0.1 h	WDT	0.1 h	WDT	0.1 h	WDT	0.1 h	WDT
Ceftiofur*	Cattle	Healthy	0.32	Indeterminant	1.15	Indeterminant	0.28	Indeterminant	0.27	Indeterminant
		Diseased	0.15	Indeterminant	0.37	Indeterminant	0.13	Indeterminant	0.12	Indeterminant
	Swine	Healthy	0.026	Indeterminant	0.079	Indeterminant	0.023	Indeterminant	0.02	Indeterminant
		Diseased	0.0089	1328579	0.026	1367886	0.008	1306966	0.007	1323341
Enrofloxacin	Cattle	Healthy	0.046	0.91	0.21	1.51	0.011	0.24	0.02	0.91
		Diseased	0.017	0.38	0.067	0.5	0.004	0.1	0.0076	0.38
	Swine	Healthy	0.0022	0.18	0.018	0.25	0.00013	0.012	0.00061	0.18
		Diseased	0.00075	0.048	0.0062	0.066	0.000044	0.003	0.00021	0.048
Flunixin	Cattle	Healthy	0.018	0.093	0.024	0.089	0.00019	0.0017	0.004	0.07
		Diseased	0.0061	0.029	0.008	0.027	0.000066	0.0005	0.0014	0.02
	Swine	Healthy	0.00074	0.012	0.0032	0.013	0.0000064	0.00026	0.00011	0.0091
		Diseased	0.00025	0.004	0.0011	0.004	0.0000021	0.000086	0.000038	0.003
Sulfamethazine	Cattle	Healthy	0.038	1.15	0.018	0.58	0.06	1.89	0.011	0.19
		Diseased	0.013	0.21	0.006	0.1	0.02	0.34	0.0037	0.035
	Swine	Healthy	0.0081	0.13	0.021	0.34	0.018	0.4	0.0068	0.13
		Diseased	0.0026	0.039	0.007	0.099	0.0059	0.12	0.0022	0.039

Note: WDT represents the withdrawal time. The withdrawal time is dependent on the formulation of the drug. In this table, M/D ratios represent the model-predicted M/D ratios at the first simulation time point (0.1 h) and at withdrawal times after exposure to labeled therapeutic regimens of studied drugs. The labeled therapeutic regimens and associated withdrawal times of these drugs are shown in Supplementary Table 9. * “Indeterminant” indicates that the model-predicted M/D ratios at the withdrawal time could not be accurately estimated (i.e., >10^20^) since the metabolic transformation for ceftiofur was so rapid that at the withdrawal time, the residue was essentially all metabolites.
