# Supplementary material for: Human Food Safety Implications of Variation in Food Animal Drug Metabolism
Source: Sci Rep. 2016 Jun 15;6:27907. doi: 10.1038/srep27907 (PMC4908408; doi:10.1038/srep27907)
Supplement: Supplementary Information [file srep27907-s1.pdf]

Supplementary Information

Human Food Safety Implications of Variation in Food Animal Drug Metabolism

Zhoumeng Lin[1], Christopher I. Vahl[2], Jim E. Riviere[1]*

[1]Institute of Computational Comparative Medicine (ICCM), Department of Anatomy and Physiology, College of Veterinary Medicine, Kansas State University, Manhattan, KS 66506, USA. [2]Department of Statistics, College of Arts and Sciences, Kansas State University, Manhattan, KS 66506, USA.

*Correspondence to: Institute of Computational Comparative Medicine, College of Veterinary Medicine, Kansas State University, 1800 Denison Avenue, P200 Mosier Hall, Manhattan, KS 66506, USA. Phone: (785) 532-3683; Fax: (785) 532-4953; Email: jriviere@ksu.edu; Website: http://www.k-state.edu/iccm/.

Contents Page

Supplementary Figures 3

Figure 1. PBPK model calibration results for ceftiofur in cattle. 3

Figure 2. PBPK model calibration results for ceftiofur in swine...4

Figure 3. PBPK model calibration results for enrofloxacin in cattle...5

Figure 4. PBPK model calibration results for enrofloxacin in swine 6

Figure 5. PBPK model calibration results for flunixin in cattle. 7

Figure 6. PBPK model calibration results for flunixin in swine..8

Figure 7. PBPK model calibration results for sulfamethazine in cattle......................................9

Figure 8. PBPK model calibration results for sulfamethazine in swine 10

Figure 9. Regression analysis results of PBPK model evaluation datasets 11

Figure 10. Comparisons of model-predicted and measured M/D ratios...................................12

Supplementary Tables..13

Table 1. Pharmacokinetic studies used in the calibration and evaluation of the PBPK model .13

Table 2. Physiological parameters used in the PBPK model...15

Table 3. Chemical-specific parameters used in the PBPK model for ceftiofur........................16

Table 4. Chemical-specific parameters used in the PBPK model for enrofloxacin..................17

Table 5. Chemical-specific parameters used in the PBPK model for flunixin.........................18

Table 6. Chemical-specific parameters used in the PBPK model for sulfamethazine..............19

Table 7. Normalized sensitivity coefficients of highly sensitive parameters 20

Table 8. Pharmacokinetic studies of selected drugs approved in food animals showing variability of M/D ratios in healthy and diseased cattle and swine 21

Table 9. Marker residues, target tissues, tolerances, and representative labeled therapeutic regimens of ceftiofur, enrofloxacin, flunixin, and sulfamethazine in cattle and swine22

PBPK Model Code 23

Supplementary References 31

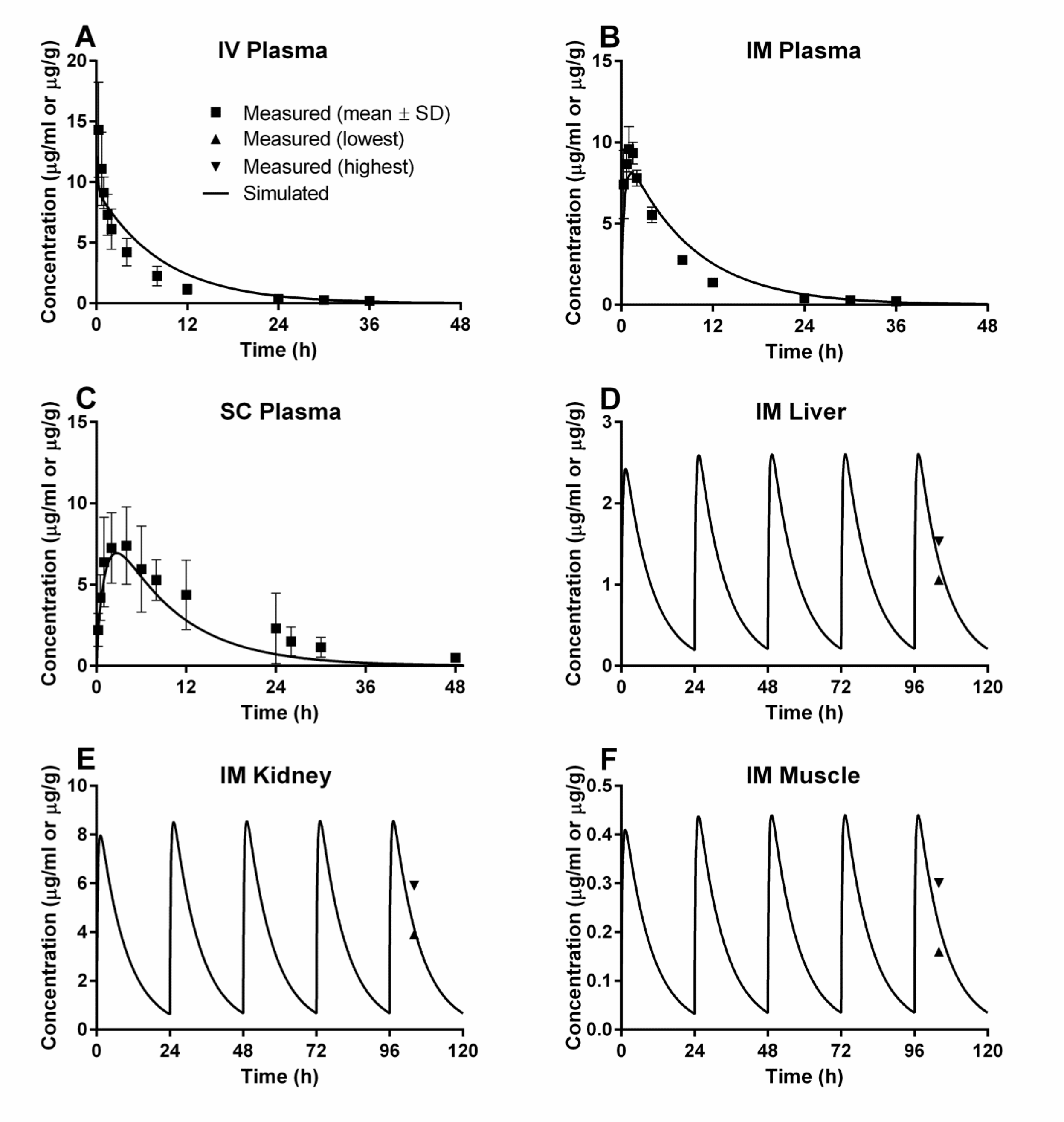

Supplementary Figure 1. **PBPK model calibration results for ceftiofur in cattle.** Comparisons of model-simulated (lines) and measured concentrations (symbols) of ceftiofur plus its major metabolite in the plasma and tissues of cattle after intravenous (IV), intramuscular (IM), or subcutaneous (SC) injection with ceftiofur. Measured data are from references (*1*), (*2*), and (*3*). Details of these references are provided in Supplementary Table 1. ■: mean ± SD/SEM; ▼: the highest measured value; ▲: the lowest measured value.

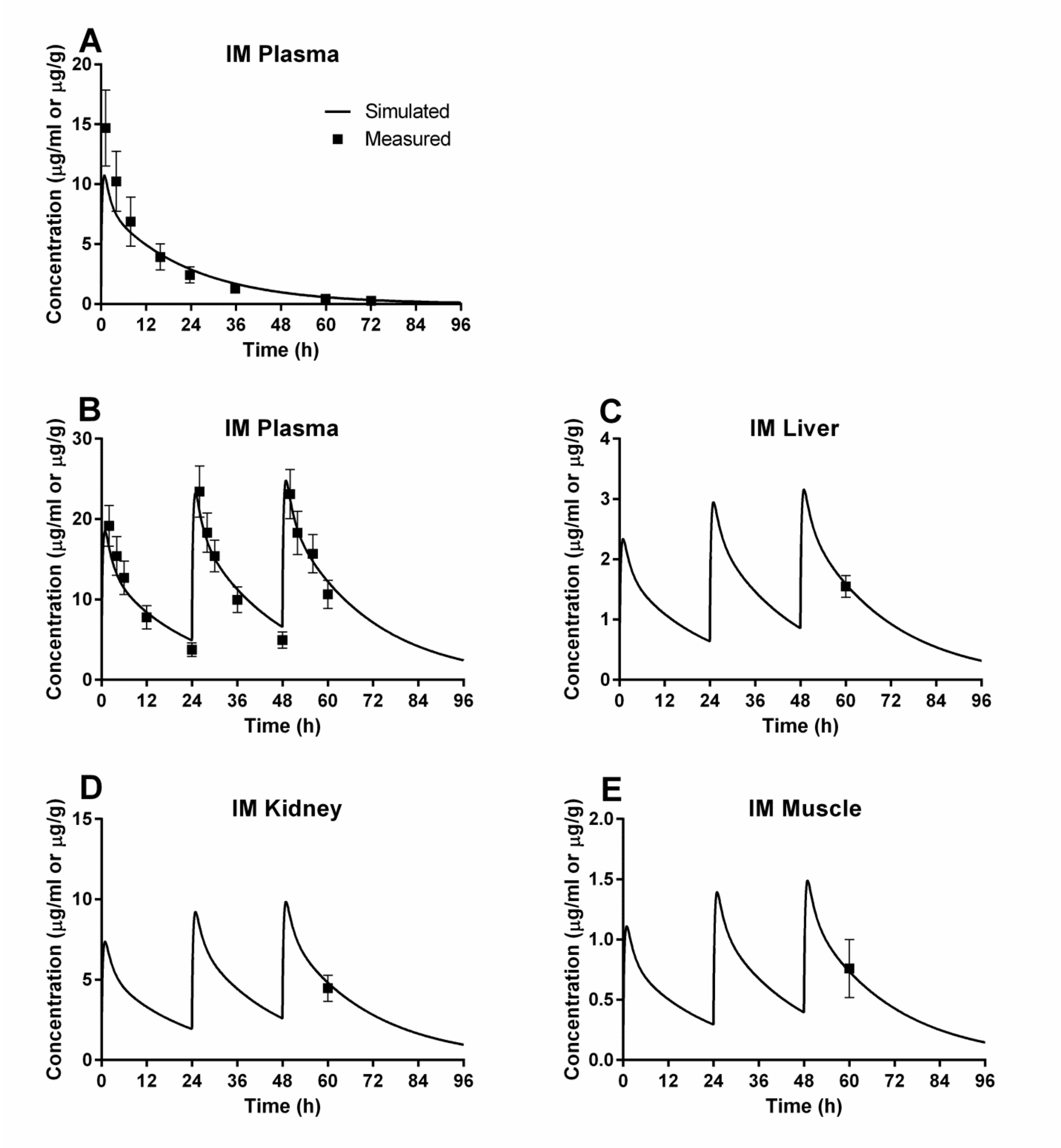

Supplementary Figure 2. **PBPK model calibration results for ceftiofur in swine.** Comparisons of model-simulated (lines) and measured concentrations (solid square symbols) of ceftiofur plus its major metabolite in the plasma and tissues of swine after intramuscular (IM) injection with ceftiofur. Measured data are from references (*4*) and (*5*). Details of these references are provided in Supplementary Table 1.

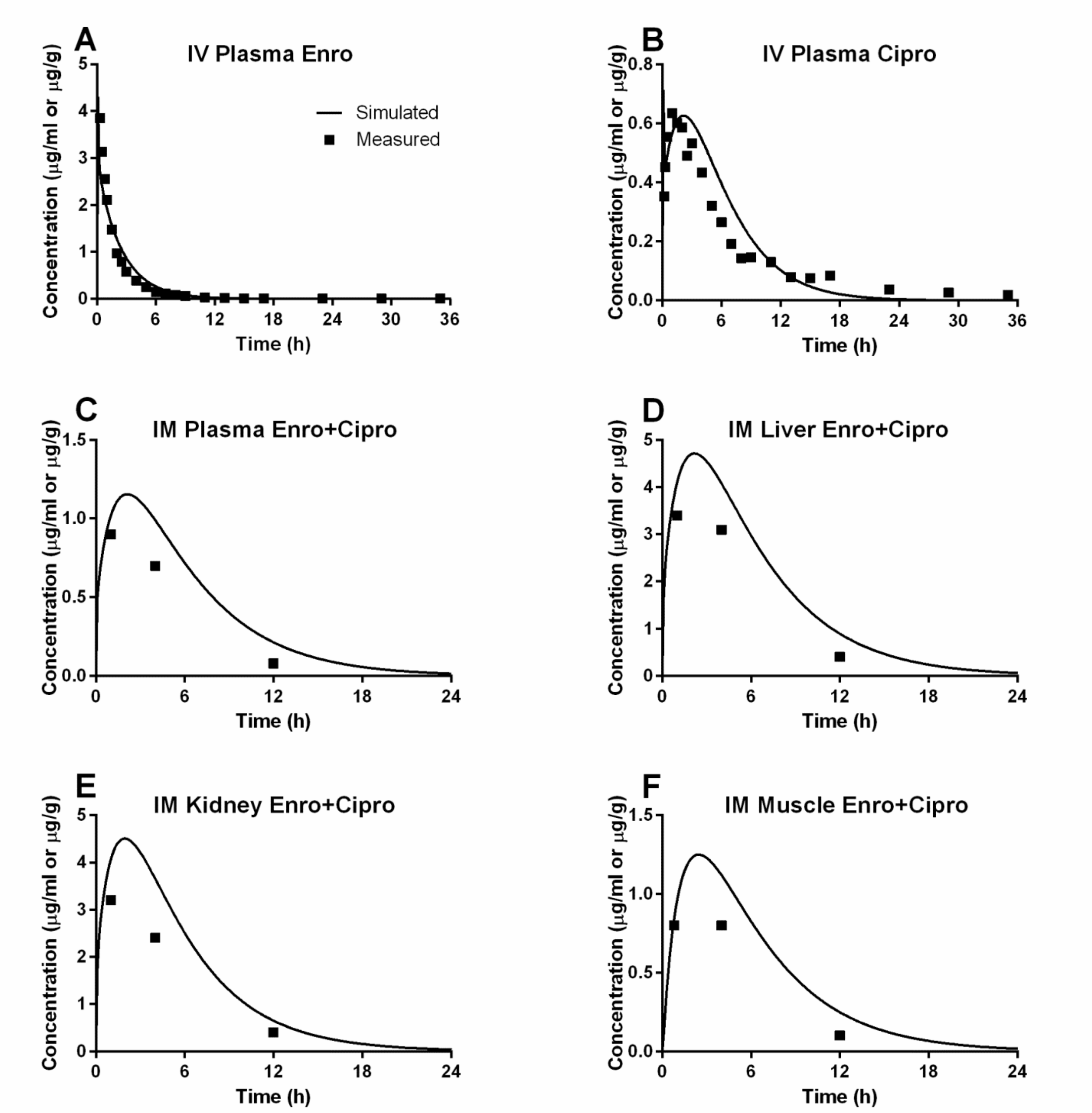

Supplementary Figure 3. **PBPK model calibration results for enrofloxacin in cattle.** Comparisons of model-simulated (lines) and measured concentrations (solid square symbols) of enrofloxacin (Enro) and/or its main metabolite ciprofloxacin (Cipro) in the plasma and tissues of cattle after intravenous (IV) or intramuscular (IM) injection with enrofloxacin. Measured data are from references (*6*) and (*7*). Details of these references are provided in Supplementary Table 1.

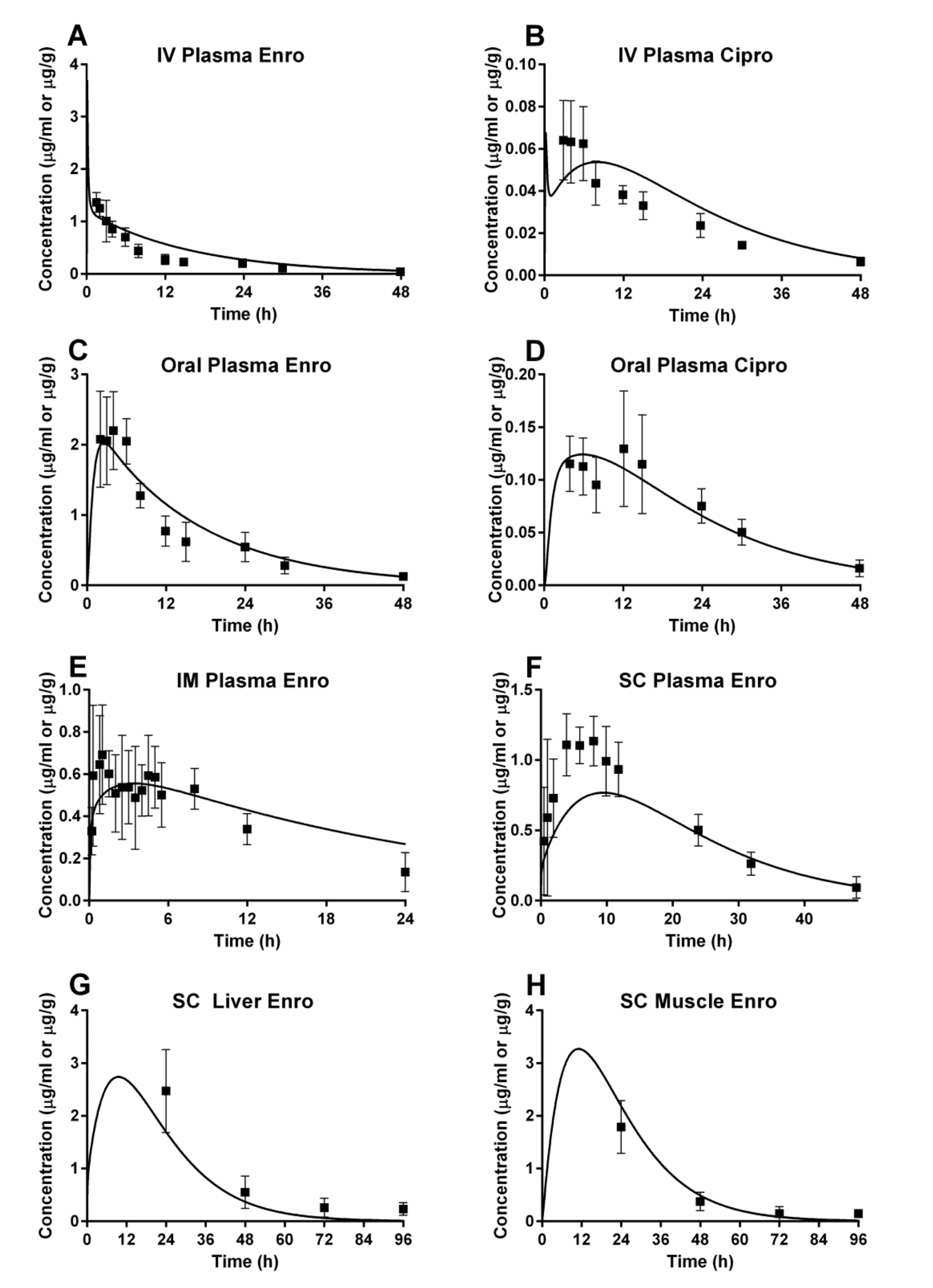

Supplementary Figure 4. **PBPK model calibration results for enrofloxacin in swine.** Comparisons of model-simulated (lines) and measured concentrations (solid square symbols) of enrofloxacin (Enro) or its major metabolite ciprofloxacin (Cipro) in the plasma and tissues of swine after intravenous (IV), intramuscular (IM), or subcutaneous (SC) injection, or oral exposure with enrofloxacin. Measured data are from references (*8*), (*9*), and (*10*). Details of these references are provided in Supplementary Table 1.

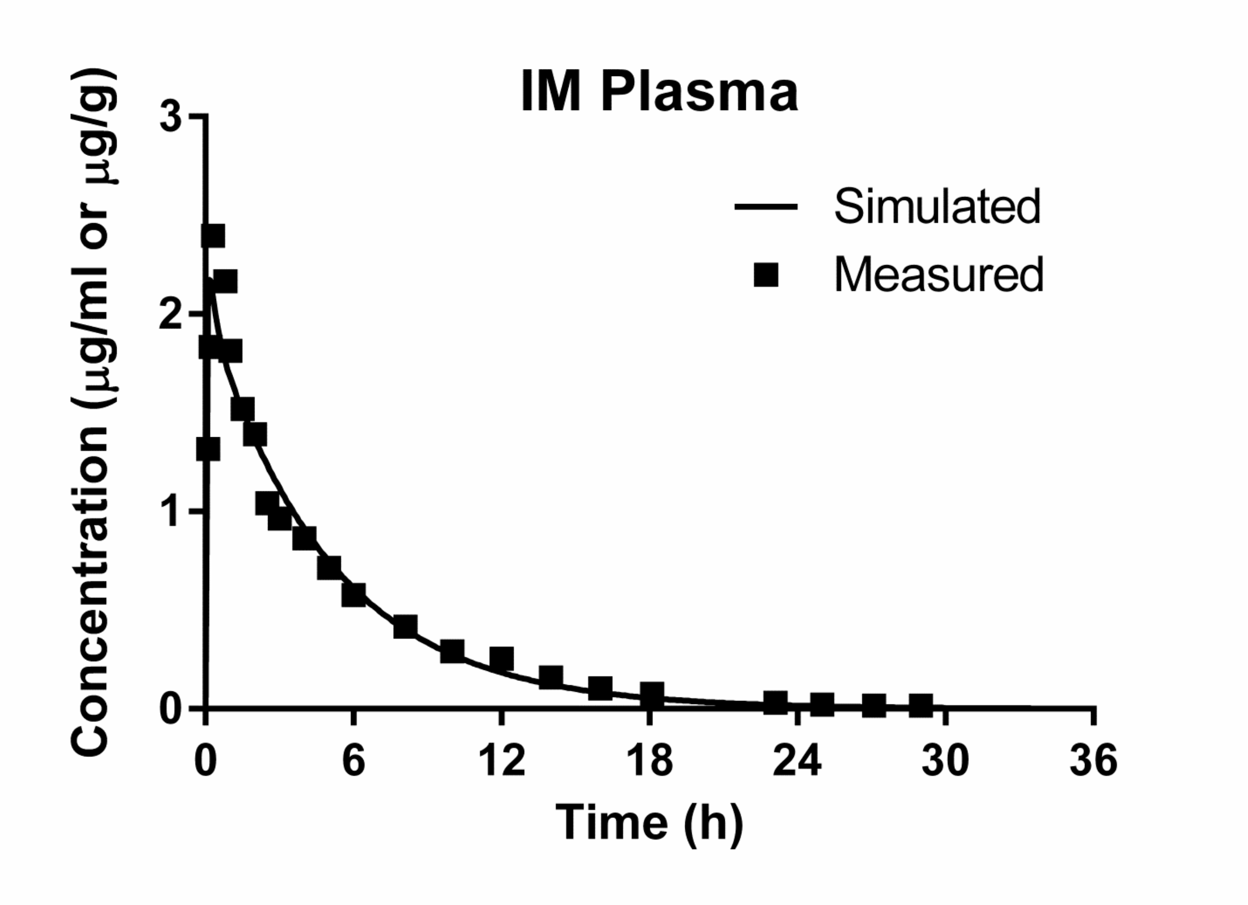

Supplementary Figure 5. **PBPK model calibration results for flunixin in cattle.** Comparisons of model-simulated (line) and measured concentrations (solid square symbols) of flunixin in the plasma of cattle after intramuscular (IM) injection. Measured data are from reference (*11*). Refer to Supplementary Table 1 for details of this reference.

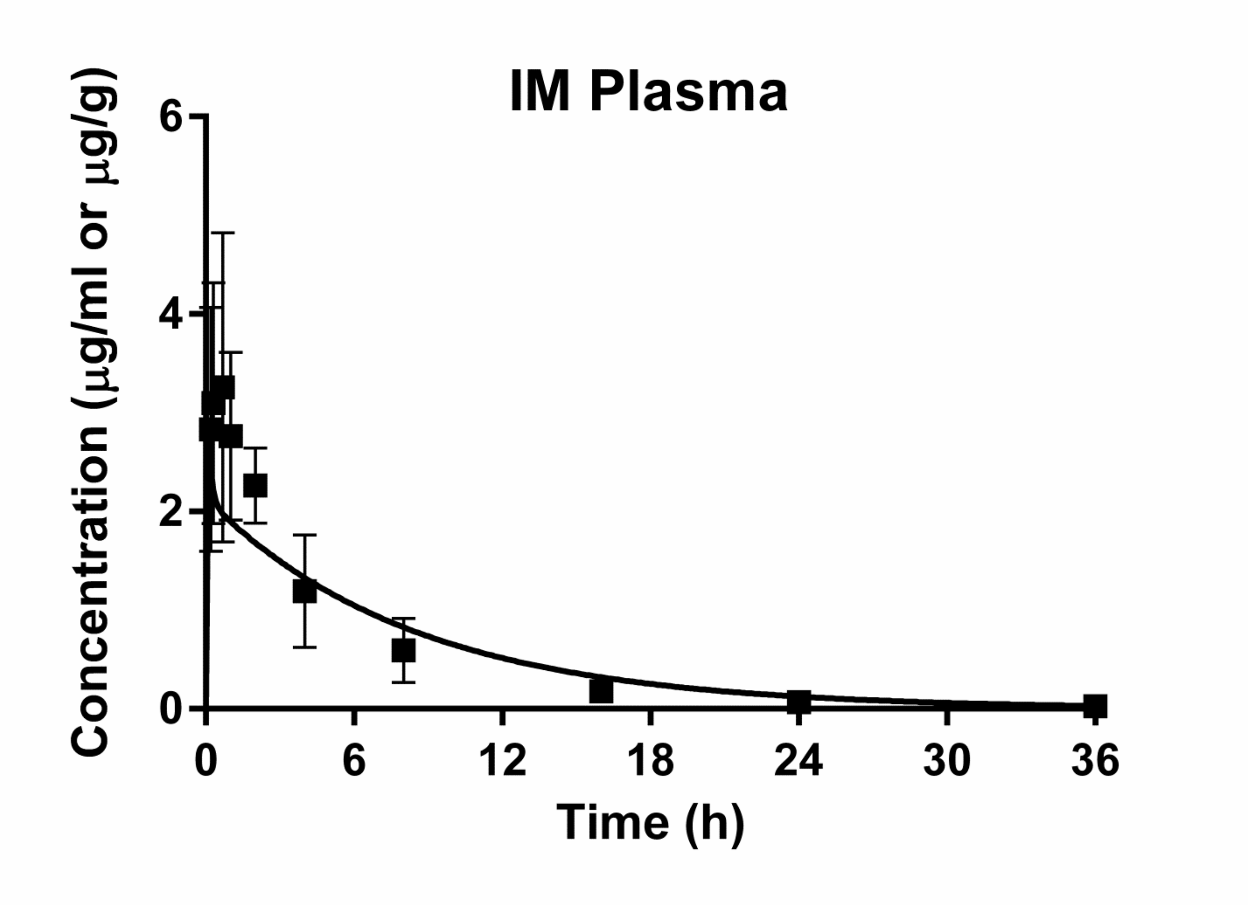

Supplementary Figure 6. **PBPK model calibration results for flunixin in swine.** Comparisons of model-simulated (line) and measured concentrations (solid square symbols) of flunixin in the plasma of swine after intramuscular (IM) injection. Measured data are from reference (*12*). Refer to Supplementary Table 1 for details of this reference.

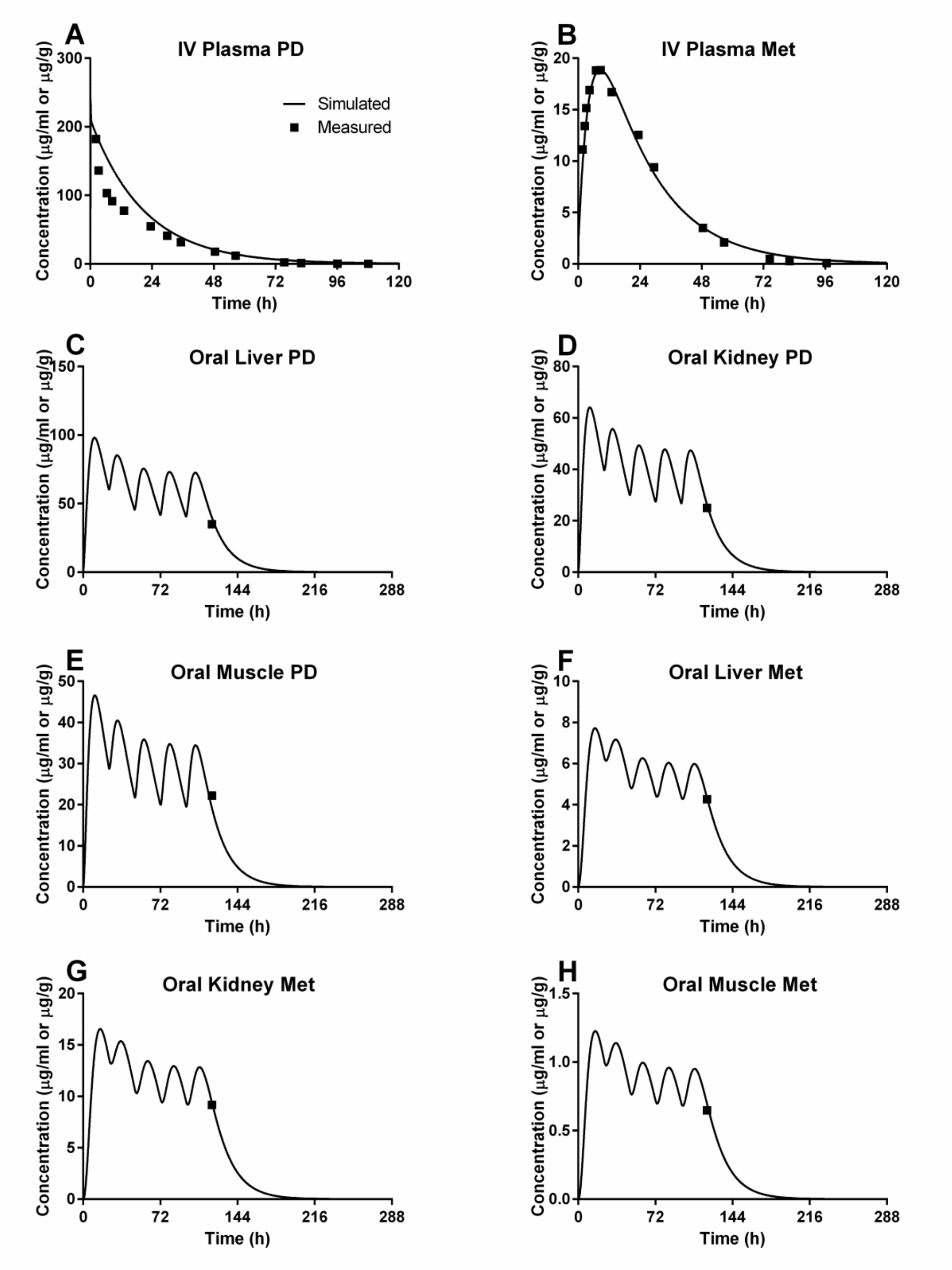

Supplementary Figure 7. **PBPK model calibration results for sulfamethazine in cattle.** Comparisons of model-simulated (lines) and measured concentrations (solid square symbols) of sulfamethazine or its major metabolite N-acetyl sulfamethazine in the plasma and tissues of cattle after intravenous injection (IV) or oral exposure with sulfamethazine. Measured data are from references (*13*) and (*14*). Refer to Supplementary Table 1 for details of these references. PD: the parent drug sulfamethazine; Met: the main metabolite N-acetyl sulfamethazine.

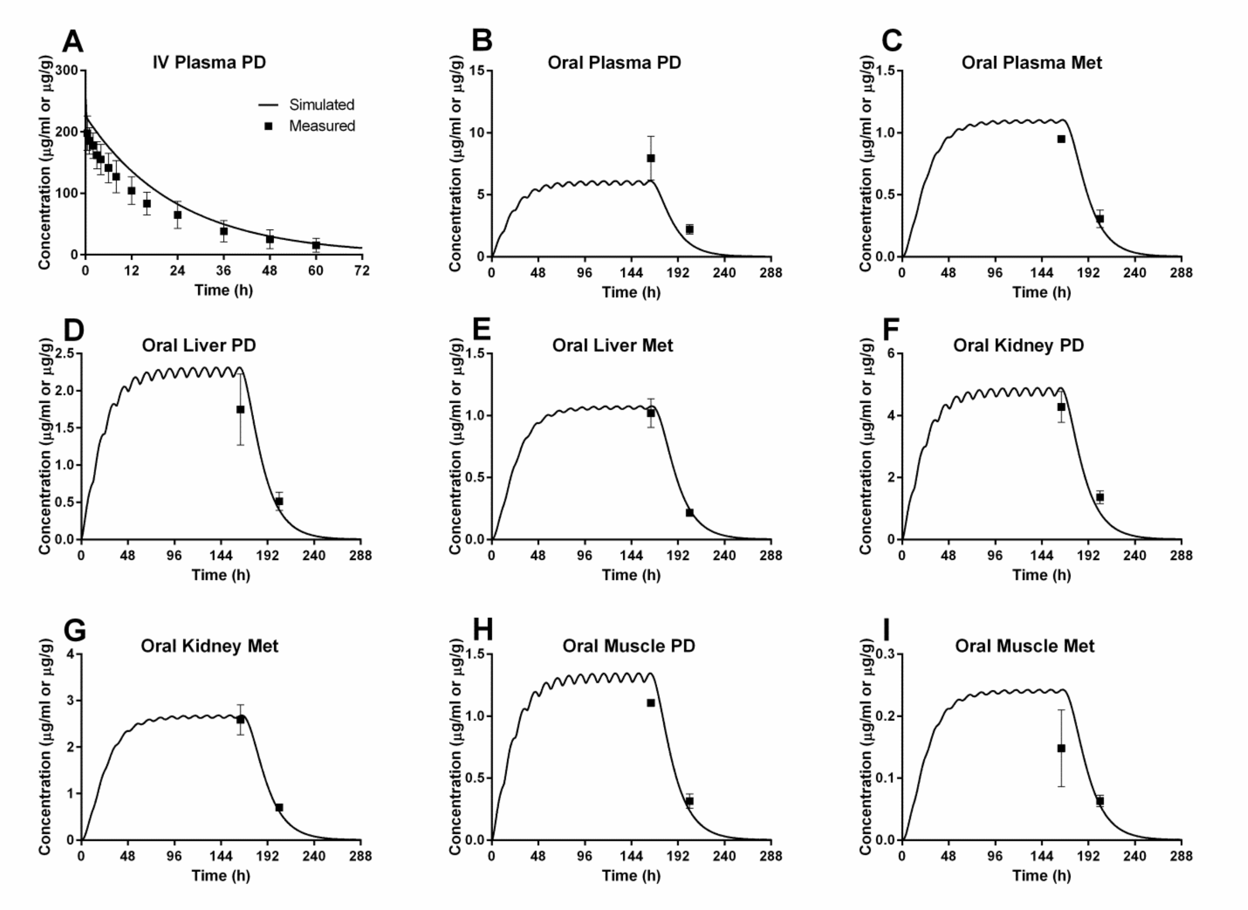

Supplementary Figure 8. **PBPK model calibration results for sulfamethazine in swine.** Comparisons of model-simulated (lines) and measured concentrations (solid square symbols) of sulfamethazine or its major metabolite N-acetyl sulfamethazine in the plasma and tissues of swine after intravenous injection (IV) or oral exposure with sulfamethazine. Measured data are from references (*15*) and (*16*). Refer to Supplementary Table 1 for details of these references. PD: the parent drug sulfamethazine; Met: the main metabolite N-acetyl sulfamethazine.

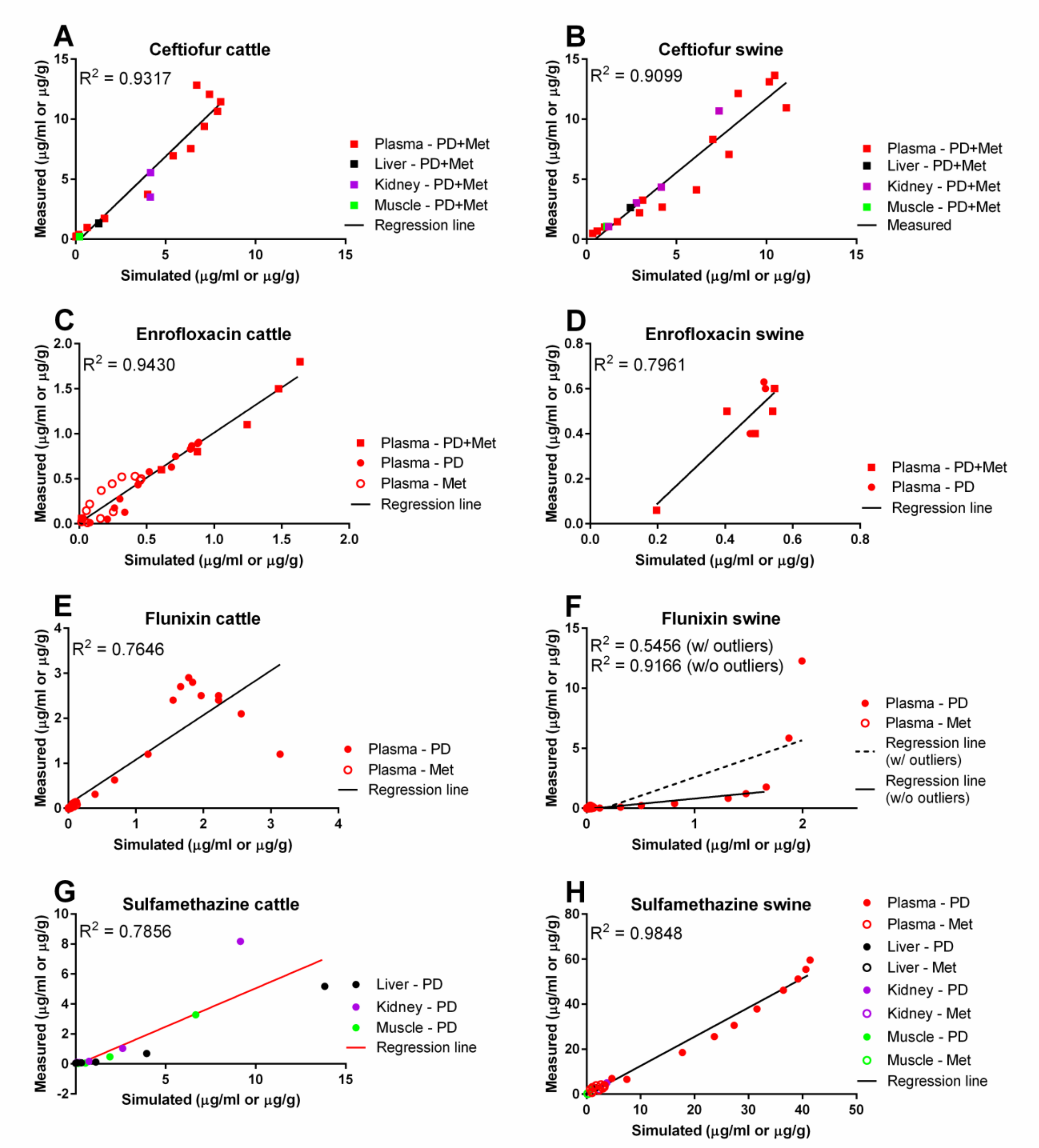

Supplementary Figure 9. Regression analysis results of PBPK model evaluation datasets. After model calibration, PBPK models for ceftiofur, enrofloxacin, flunixin, and sulfamethazine in healthy cattle and swine were further evaluated based on datasets listed in Supplementary Table 1. Each panel represents the result of a regression analysis between measured and model-simulated plasma or tissue drug concentrations for each drug in each species. PD: concentrations of the parent drug; Met: concentrations of the main metabolite; PD+Met: concentrations of parent drug plus its major metabolite. R^2 values and regression lines are provided in each panel.

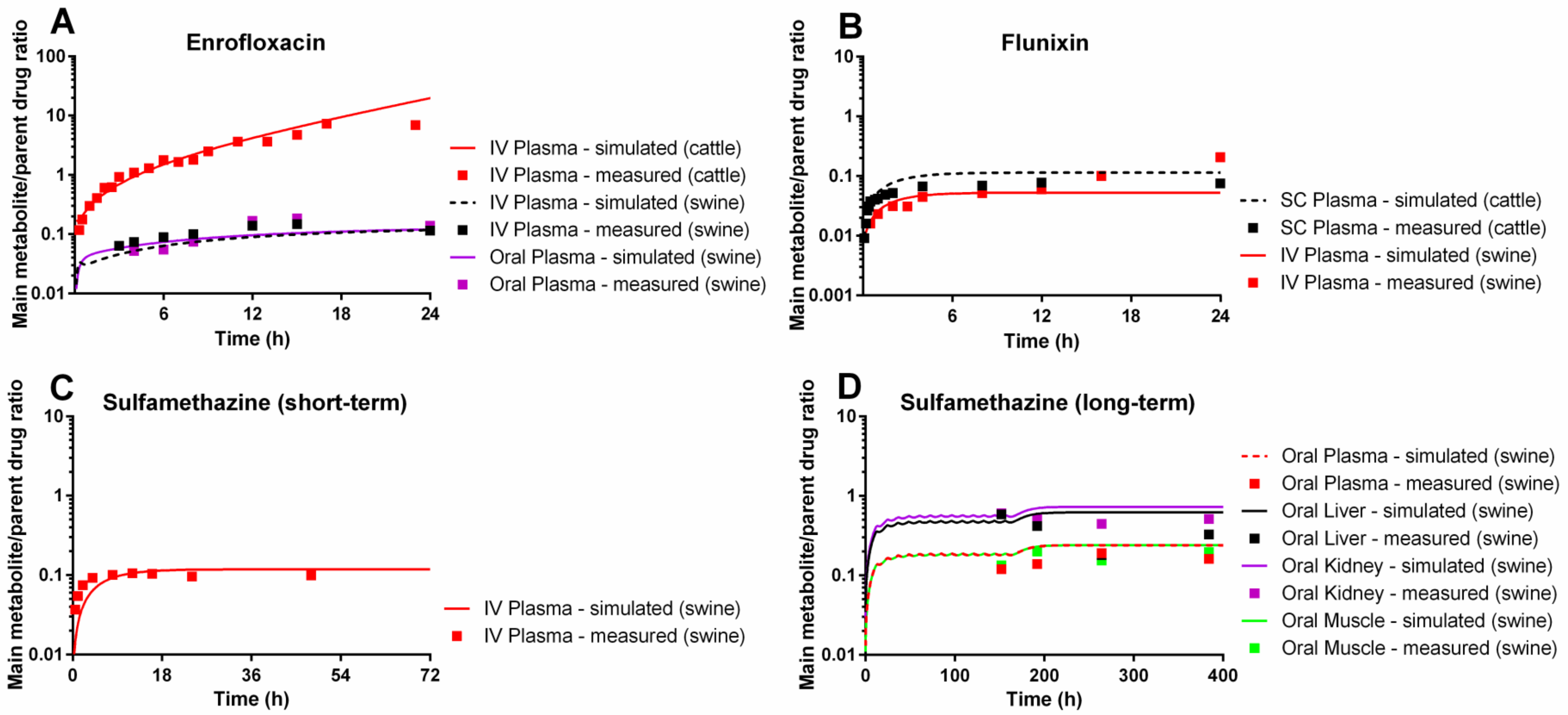

Supplementary Figure 10. **Comparisons of model-predicted and measured main metabolite to parent drug (M/D) ratios.** M/D ratios for enrofloxacin (A), flunixin (B) and sulfamethazine (C, D) in the plasma or tissues of healthy cattle and swine after oral exposure, intravenous (IV) or subcutaneous (SC) injection are shown. Experimental data are from references (*7*) and (*8*) for enrofloxacin, from references (*17*) and (*18*) for flunixin, and from references (*16*) and (*18*) for sulfamethazine.

Supplementary Table 1. Pharmacokinetic studies used in the calibration and evaluation of the PBPK model for ceftiofur, enrofloxacin, flunixin, and sulfamethazine in cattle and swine.

Drug/Purpose/Route	Dose (mg/kg)	Species	Sex	n	BW (kg)	Age	Matrix	Assay	Ref.
Ceftiofur, calibration									
IV, IM	2.2	Cattle	M+F	7	217-276	6 months	P	HPLC	(*2*)
SC	2.2	Cattle	F	6	149-269	6 months	P	HPLC	(*1*)
IM	2.2	Cattle	M+F	NA	124.5-192.5	NA	L, K, Mu	Radioactivity	(*3*)
IM	3	Swine	M+F	12	28-77.6	3-4 months	P	HPLC	(*5*)
IM	5	Swine	M+F	12	33-47	NA	P, L, K, Mu	Radioactivity	(*4*)
Ceftiofur, evaluation									
IM	2.2	Cattle	M+F	6	NA	NA	P	HPLC	(*19*)
IM	2.2	Cattle	NA	NA	NA	NA	L, K, Mu	Radioactivity	(*3*)
IM	2.2	Cattle	NA	NA	NA	NA	L, K, Mu	Radioactivity	(*20*)
IM	3	Swine	NA	3	21-23.7	NA	P, K	HPLC	(*21*)
IM	3	Swine	NA	8	NA	1 month	P	HPLC	(*22*)
IM	7.5	Swine	M+F	24	25-40	3-6 months	L, K, Mu	Radioactivity	(*23*)
Enrofloxacine, calibration									
IV	5	Cattle	M	6	159-213	NA	P	HPLC	(*7*)
IM	2.5	Cattle	NA	6	62-94	NA	P, L, K, Mu	Agar diffusion test	(*6*)
IV, oral	5-10	Swine	NA	8	25-40	3-4 months	P	HPLC	(*8*)
IM	2.5	Swine	M+F	12	8.5-12.2	2 months	P	HPLC	(*9*)
SC	7.5	Swine	M+F	14	52.5-64.5	4 months	P, L, Mu	TFC-MS/MS	(*10*)

Supplementary Table 1. Continued

Drug/Purpose/ Route	Dose (mg/kg)	Species	Sex	n	BW (kg)	Age	Matrix	Assay	Ref.
Enrofloxacin, evaluation									
IV	2.5	Cattle	NA	6-10	43-76	<2 months	P	Agar diffusion test	(*6*)
SC	12.5	Cattle	NA	5	182-205	4-5 months	P	HPLC	(*24*)
Oral	2.5	Swine	M+F	17	12.5-31	NA	P	Agar diffusion test	(*6*)
IM	2.5	Swine	F	2-6	24-31	2 months	P	HPLC	(*25*)
Flunixin, calibration									
IM	2.2	Cattle	F	1	462±36	NA	P	HPLC	(*11*)
IM	2.2	Swine	F	6	152-168	42 weeks	P	HPLC-MS	(*12*)
Flunixin, evaluation									
SC	2.2	Cattle	M+F	8	258-340	8-9 months	P	LC-MS/MS	(*17*)
IV	2.2	Swine	M+F	8-21	40.15	10-14 weeks	P	UPLC-MS/MS	(*18*)
Sulfamethazine, calibration									
IV	100	Cattle	M	3	70-77	2 months	P	HPLC	(*13*)
Oral	110-220	Cattle	M	4	75-114	3 months	L, K, Mu	GC	(*14*)
IV	107.5	Swine	M	8	47	12-13 weeks	P	Bratton-Marshall	(*15*)
Oral	2.5	Swine	M+F	3	60-70	NA	P, L, K, Mu	Radioacitivity & HPLC	(*16*)
Sulfamethazine, evaluation									
Oral	110-220	Cattle	M+F	4	47.6-113.4	NA	L, K, Mu	Bratton-Marshall	(*26*)
IV	20	Swine	M+F	7-20	40.15	2-4 months	P	HPLC	(*18*)
Oral	2.2	Swine	M+F	3	71-79	NA	P, L, K, Mu	Radioacitivity & HPLC	(*27*)

Note: IM, intramuscular; IV, intravenous; SC, subcutaneous; NA, not available; F, female; M, male; P, plasma; L, liver; K, kidney; Mu, muscle; Ref., reference.

Supplementary Table 2. Physiological parameters used in the PBPK model for ceftiofur, enrofloxacin, flunixin, and sulfamethazine in cattle and swine.

Parameter	Abbreviation	Cattle	Swine	Reference
Body weight (kg)	BW	250	40	Study-specific
Cardiac output (L/h/kg)	QCC	5.67	5	(*28*-*30*)
Tissue volume (fraction of body weight, unitless)				
Arterial blood	VartC	0.0104	0.0156	(*28*-*30*)
Venous blood	VvenC	0.0296	0.0444	(*28*-*30*)
Liver	VLC	0.013	0.0247	(*28*-*30*)
Kidney	VKC	0.0035	0.004	(*28*-*30*)
Muscle	VMC	0.27	0.4	(*28*-*30*)
Fat	VFC	0.15	0.32	(*28*-*30*)
Lung	VLuC	0.008	0.01	(*28*-*30*)
Rest of body	VrestC	0.5155	0.1813	(*28*-*30*)
Blood flow (fraction of cardiac output, unitless)				
Liver	QLC	0.35	0.2725	(*28*, *29*, *31*)
Kidney	QKC	0.09	0.12	(*28*-*30*)
Muscle	QMC	0.18	0.251	(*28*-*30*)
Fat	QFC	0.08	0.1275	(*28*-*30*)
Rest of body	QrestC	0.3	0.229	(*28*-*30*)

Note: All swine physiological parameter values represent the average of values from references (*28*) and (*29*), except QCC that is from reference (*29*). All cattle physiological parameters are the same as those in the flunixin PBPK model in cattle (*30*), except VLC that is from reference (*31*).

Supplementary Table 3. Chemical-specific parameters used in the PBPK model for ceftiofur in cattle and swine.

Parameter	Abbreviation	Cattle	Swine
Absorption rate constant (/h)			
Gastric emptying	Kst	NA	NA
Intestinal absorption	Ka	NA	NA
Intramuscular	Kim	2	3
Subcutaneous	Ksc	0.8	NA
Tissue:plasma partition coefficient for the parent drug (unitless)			
Liver	PL	0.3	0.13
Kidney	PK	1	0.4
Muscle	PM	0.05	0.06
Fat	PF	0.05	0.05
Lung	Plu	0.2	0.2
Rest of body	Prest	0.1	0.5
Tissue:plasma partition coefficient for the main metabolite (unitless)			
Liver	PL1	0.3	0.13
Kidney	PK1	1	0.4
Muscle	PM1	0.05	0.06
Fat	PF1	0.05	0.05
Lung	Plu1	0.2	0.2
Rest of body	Prest1	0.1	0.5
*Hepatic metabolic rate [/(h*kg)]*	KmC	2	1
Fraction of parent drug metabolized to the main metabolite (unitless)	Frac	0.7	0.7
Percentage of plasma protein binding (unitless)			
Parent drug	PB	0.58	0.58
Main metabolite	PB1	0.58	0.58
Fecal elimination rate constant (/h)	Kfeces	NA	NA
Urinary elimination rate constant (L/h/kg)			
Parent drug	KurineC	0.0016	0.001
Main metabolite	Kurine1C	0.016	0.01

NA: not applicable.

Supplementary Table 4. Chemical-specific parameters used in the PBPK model for enrofloxacin in cattle and swine.

Parameter	Abbreviation	Cattle	Swine
Absorption rate constant (/h)			
Gastric emptying	Kst	NA	1
Intestinal absorption	Ka	NA	1
Intramuscular	Kim	0.7	0.5
Subcutaneous	Ksc	0.06	0.1
Tissue:plasma partition coefficient for the parent drug (unitless)			
Liver	PL	4.3	4.3
Kidney	PK	5.5	5.5
Muscle	PM	1.09	3
Fat	PF	0.53	0.53
Lung	Plu	4.3	4.3
Rest of body	Prest	1.5	8
Tissue:plasma partition coefficient for the main metabolite (unitless)			
Liver	PL1	4.3	4.3
Kidney	PK1	5.5	5.5
Muscle	PM1	1.09	4.3
Fat	PF1	0.53	0.53
Lung	Plu1	4.3	4.3
Rest of body	Prest1	1.5	8
*Hepatic metabolic rate [/(h*kg)]*	KmC	0.06	0.045
Fraction of parent drug metabolized to the main metabolite (unitless)	Frac	0.55	0.35
Percentage of plasma protein binding (unitless)			
Parent drug	PB	0.46	0.46
Main metabolite	PB1	0.19	0.19
Fecal elimination rate constant (/h)	Kfeces	NA	0.01
Urinary elimination rate constant (L/h/kg)			
Parent drug	KurineC	0.15	0.12
Main metabolite	Kurine1C	1.99	10

NA: not applicable.

Supplementary Table 5. Chemical-specific parameters used in the PBPK model for flunixin in cattle and swine.

Parameter	Abbreviation	Cattle	Swine
Absorption rate constant (/h)			
Gastric emptying	Kst	NA	NA
Intestinal absorption	Ka	NA	NA
Intramuscular	Kim	5	10
Subcutaneous	Ksc	10	10
Tissue:plasma partition coefficient for the parent drug (unitless)			
Liver	PL	1.35	1.35
Kidney	PK	1.35	1.35
Muscle	PM	0.665	0.665
Fat	PF	0.503	0.503
Lung	Plu	2.16	2.16
Rest of body	Prest	1	1
Tissue:plasma partition coefficient for the main metabolite (unitless)			
Liver	PL1	1	1
Kidney	PK1	1	1
Muscle	PM1	0.5	0.5
Fat	PF1	0.5	0.5
Lung	Plu1	1	1
Rest of body	Prest1	0.5	0.5
*Hepatic metabolic rate [/(h*kg)]*	KmC	0.03	0.025
Fraction of parent drug metabolized to the main metabolite (unitless)	Frac	0.3	0.2
Percentage of plasma protein binding (unitless)			
Parent drug	PB	0.95	0.95
Main metabolite	PB1	0.95	0.95
Fecal elimination rate constant (/h)	Kfeces	NA	NA
Urinary elimination rate constant (L/h/kg)			
Parent drug	KurineC	0.001	0.001
Main metabolite	Kurine1C	20	20

NA: not applicable.

Supplementary Table 6. Chemical-specific parameters used in the PBPK model for sulfamethazine in cattle and swine.

Parameter	Abbreviation	Cattle	Swine
Absorption rate constant (/h)			
Gastric emptying	Kst	0.5	0.15
Intestinal absorption	Ka	0.2	0.2
Intramuscular	Kim	NA	NA
Subcutaneous	Ksc	NA	NA
Tissue:plasma partition coefficient for the parent drug (unitless)			
Liver	PL	0.378	0.378
Kidney	PK	0.25	0.8
Muscle	PM	0.18	0.22
Fat	PF	0.336	0.336
Lung	Plu	0.378	0.378
Rest of body	Prest	0.6	1
Tissue:plasma partition coefficient for the main metabolite (unitless)			
Liver	PL1	0.18	0.9
Kidney	PK1	0.5	3
Muscle	PM1	0.03	0.22
Fat	PF1	0.336	0.336
Lung	Plu1	0.378	0.378
Rest of body	Prest1	0.6	1
*Hepatic metabolic rate [/(h*kg)]*	KmC	0.05	0.046
Fraction of parent drug metabolized to the main metabolite (unitless)	Frac	0.65	0.65
Percentage of plasma protein binding (unitless)			
Parent drug	PB	0.57	0.57
Main metabolite	PB1	0.57	0.57
Fecal elimination rate constant (/h)	Kfeces	0.01	0.05
Urinary elimination rate constant (L/h/kg)			
Parent drug	KurineC	0.005	0.0001
Main metabolite	Kurine1C	0.12	0.14

NA: not applicable.

Supplementary Table 7. Normalized sensitivity coefficients of highly sensitive parameters on selected key dose metrics.

Parameter	Parent drug				Main metabolite			
	AUCCV	AUCCL	AUCCK	AUCCM	AUCCV	AUCCL	AUCCK	AUCCM
QCC	-0.10	0.02	0.10	-0.09	-0.63	-0.74	0.31	-0.56
QKC	-0.07	-0.07	0.13	-0.07	-0.72	-0.50	0.22	-0.70
BW	-0.37	-0.49	-0.37	-0.36	0.55	0.54	0.55	0.56
VLC	-0.37	-0.49	-0.37	-0.36	0.56	0.54	0.56	0.58
PL	-0.38	0.49	-0.38	-0.38	0.54	0.52	0.54	0.55
PK	0.00	0.00	1.00	0.00	0.00	0.00	0.00	0.00
PM	-0.11	-0.11	-0.11	0.87	-0.14	-0.13	-0.14	-0.15
PL1	0.00	0.00	0.00	0.00	-0.01	0.99	-0.01	-0.01
PK1	0.00	0.00	0.00	0.00	0.00	0.00	1.00	0.00
PM1	0.00	0.00	0.00	0.00	-0.11	-0.07	-0.11	0.84
KmC	-0.38	-0.50	-0.38	-0.37	0.55	0.53	0.55	0.56
Frac	0.00	0.00	0.00	0.00	1.00	1.00	1.00	1.00
Kurine1C	0.00	0.00	0.00	0.00	-0.04	-0.03	-0.97	-0.04

Note: Only parameters with at least one absolute value of NSC greater than 0.5 are presented. AUCCV, AUCCL, AUCCK, and AUCCM represent 24-h area under concentration curves of parent drug or main metabolite in the plasma, liver, kidney, and muscle, respectively. Detailed description of parameters refers to Supplementary Tables 2-6.

Supplementary Table 8. Pharmacokinetic studies of selected drugs approved in food animals showing variability of main metabolite to parent drug (M/D) ratios in healthy and diseased cattle and swine.

Drug/Route	Species	Status	Dose (mg/kg)	n	Matrix	Assay	M/D ratio	Time points (h)	Ref.
Enrofloxacin									
IV	Cattle	Healthy	5	6	P	HPLC	0.12-7.33	0.33-35	(*7*)
SC	Cattle	Healthy	12.5	5	P	HPLC	0.12-1.12	0.2-48	(*24*)
IV	Cattle	Diseased	5	6	P	HPLC	0.02-1.95	0.03-12	(*32*)
SC	Cattle	Diseased	5	6	P	HPLC	0.44-1.09	0.03-72	(*32*)
IV	Swine	Healthy	5	8	P	HPLC	0.06-0.16	3-48	(*8*)
Oral	Swine	Healthy	10	8	P	HPLC	0.05-0.19	4-48	(*8*)
SC	Swine	Healthy	7.5	6	P	HPLC	0.04-0.07	0.6-48	(*33*)
Sulfamethazine									
IV	Cattle	Healthy	100	3	P	HPLC	0.06-0.26	2-96	(*13*)
Oral	Cattle	Healthy	110-220	4	L	GC	0.12-0.66	24-240	(*14*)
Oral	Cattle	Healthy	110-220	4	K	GC	0.37-0.98	24-240	(*14*)
Oral	Cattle	Healthy	110-220	4	M	GC	0.03-0.5	24-240	(*14*)
IV	Swine	Healthy	20	7-20	P	HPLC	0.03-0.1	0.5-48	(*18*)
Feed	Swine	Healthy	2.5	3	P	HPLC	0.12-0.19	8-240	(*16*)
Feed	Swine	Healthy	2.5	3	L	HPLC	0.18-0.58	8-240	(*16*)
Feed	Swine	Healthy	2.5	3	K	HPLC	0.44-0.61	8-240	(*16*)
Feed	Swine	Healthy	2.5	3	M	HPLC	0.13-0.2	8-240	(*16*)
Feed	Swine	Healthy	2.2	3	P	HPLC	0.15	8	(*27*)
Feed	Swine	Healthy	2.2	3	L	HPLC	0.36	8	(*27*)
Feed	Swine	Healthy	2.2	3	K	HPLC	0.43	8	(*27*)
Feed	Swine	Healthy	2.2	3	M	HPLC	0.1	8	(*27*)
IV	Swine	Diseased	50	7	P	HPLC	0.03-0.08	1-36	(*34*)
Flunixin									
IV	Cattle	Healthy	2.2	8	P	UHPLC-MS/MS	0.02-0.09	0.1-24	(*17*)
SC	Cattle	Healthy	2.2	8	P	UHPLC-MS/MS	0.008-0.07	0.1-24	(*17*)
IV	Swine	Healthy	2.2	8-21	P	UHPLC-MS/MS	0.02-0.13	0.5-48	(*18*)

Note: IV, intravenous; SC, subcutaneous; P, plasma; L, liver; K, kidney; M, muscle; M/D ratio, main metabolite to parent drug ratio.

Supplementary Table 9. Marker residues, target tissues, tolerances, and representative labeled therapeutic regimens of ceftiofur, enrofloxacin, flunixin, and sulfamethazine in cattle and swine.

Species/drug	Marker residue	Target tissue	Tolerance (µg/g or ppm)	Representative labeled therapeutic regimen* (Dose level X administration times, exposure route, withdrawal time)
Cattle, beef, all use classes				
Ceftiofur	Desfuroylceftiofur	Kidney	0.25	2.2 X 5, IM, 3 days
Enrofloxacin	Desethylene ciprofloxacin	Liver	0.1	7.5 X 1, SC, 28 days,
Flunixin	Flunixin free acid	Liver	0.125	2.2 X 1, IV, 4 days
Sulfamethazine	Sulfamethazine	Edible tissues	0.1	220 X 1 + 110 X 4, oral, 11 days
Swine, all use classes				
Ceftiofur	Desfuroylceftiofur	Kidney	0.4	5 X 3, IM, 4 days
Enrofloxacin	Enrofloxacin	Liver	0.5	7.5 X 1, SC, 5 days
Flunixin	Flunixin free acid	Liver	0.03	2.2 X 1, IM, 12 days
Sulfamethazine	Sulfamethazine	Edible tissues	0.1	Dose level not available. Assume the same as in cattle, oral, 15 days

Note: Data are from the VetGRAM (Veterinarian's Guide to Residue Avoidance Management) in the Food Animal Residue Avoidance Databank (FARAD). IM: intramuscular; IV: intravascular; SC, subcutaneous. * For repeated exposure paradigms, the administration interval is 24 h. The unit of the dose level is mg/kg.

PBPK Model Code

Note: The model code below represents a general physiologically based pharmacokinetic (PBPK) model for ceftiofur, enrofloxacin, flunixin, and sulfamethazine in cattle and swine. Parameter values included in the model are specifically for sulfamethazine in swine. Parameter values for all studied drugs in cattle and swine are provided in Supplementary Tables 2-6.

```
PROGRAM

INITIAL
! code that is executed once at the beginning of a simulation run goes here

!! Physiological parameters
! Blood flow rates (fraction of cardiac output)
CONSTANT QCC = 5 ! Cardiac output index (L/h/kg), also blood flow of lung, from Upton
(2008)
CONSTANT QLC = 0.2725 ! liver, average from Buur et al. (2005) and Upton (2008)
CONSTANT QKC = 0.12 ! kidney, average from Buur et al. (2005) and Upton (2008)
CONSTANT QMC = 0.251 ! Muscle, average from Buur et al. (2005) and Upton (2008)
CONSTANT QFC = 0.1275 ! Fat, average from Buur et al. (2005) and Upton (2008)

! Tissue volumes (fraction of body weight)
CONSTANT BW = 40 ! Kg, body weight was study-specific; This value was used if BW was
not given in a specific study
CONSTANT VLC = 0.0247 ! liver, average from Buur et al. (2005) and Upton (2008)
CONSTANT VKC = 0.004 ! Kidneys, average from Buur et al. (2005) and Upton (2008)
CONSTANT VMC = 0.40 ! Muscle, average from Buur et al. (2005) and Upton (2008)
CONSTANT VFC = 0.32 ! Fat, adipose tissue, average from Buur et al. (2005) and Upton
(2008)
CONSTANT VLuC = 0.01 ! Lungs, average from Buur et al. (2005) and Upton (2008)
CONSTANT VBloodC = 0.06 ! Blood, average from Buur et al. (2005) and Upton (2008)

!! Mass transfer parameters (Chemical-specific parameters)
! Chemical molecular weights and unit conversion factors, from PubChem
CONSTANT MW = 278.33 ! g/mol, sulfamethazine
CONSTANT MW1 = 320.37 ! g/mol, N-acetyl sulfamethazine
CONSTANT MWmol = 3.59 ! umol/mg, sulfamethazine, from mg to umol
CONSTANT MWmg =  0.28 ! mg/umol, sulfamethazine, from umol to mg
CONSTANT MW1mol = 3.12 ! umol/mg, N-acetyle sulfamethazine, from mg to umol
CONSTANT MW1mg = 0.32 ! mg/umol, N-acetyl sulfamethazine, from umol to mg

! Kinetic constants
! Oral absorption and fecal elimination rate constants (for parent compound)
CONSTANT Kst = 0.15 ! /h, gastric emptying rate constant
CONSTANT Ka = 0.2 ! /h, intestinal absorption rate constant
```

CONSTANT Kfeces = 0.05 ! /h, intestinal transit rate constant (fecal elimination rate constant)

! IV infusion/injection rate constants
CONSTANT Timeiv = 0.01 ! h, IV infusion/injection time

! IM absorption rate constants (set parameter value equal to 0.0 when not used in a particular simulation)
CONSTANT Kim = 0.0 ! /h, intramuscular absorption rate constant

! SC absorption rate constants
CONSTANT Ksc = 0.0 ! /h, subcutenous absorption rate constant

! Partition coefficients for parent compounds (PC, unitless)
! The values from Buur et al. 2005 were used as initial values for further estimation.
CONSTANT PL = 0.378 ! Liver:plasma PC
CONSTANT PK = 0.8 ! Kidney:plasma PC
CONSTANT PM = 0.22 ! Muscle:plasma PC
CONSTANT PF = 0.336 ! Fat:plasma PC
CONSTANT PLu = 0.378 ! Lung:plasma PC
CONSTANT Prest = 1 ! Rest-of-body:plasma PC

! Partition coefficients for the major metabolite (usually designated as the marker residue) (PC, unitless)
CONSTANT PL1 = 0.9 ! Liver:plasma PC
CONSTANT PK1 = 3 ! Kidney:plasma PC
CONSTANT PM1 = 0.22 ! Muscle:plasma PC
CONSTANT PF1 = 0.336 ! Fat:plasma PC
CONSTANT PLu1 = 0.378 ! Lung:plasma PC
CONSTANT Prest1 = 1 ! Rest-of-body:plasma PC

! Percentage plasma protein binding (unitless), Buur et al. (2005)
CONSTANT PB = 0.57 ! Percentage of parent compound bound to plasma proteins
CONSTANT PB1 = 0.57 ! Percentage of the marker residue bound to plasma proteins

! Metabolic rate constants
CONSTANT KmC = 0.046 ! /(h*kg), liver metabolic rate constant of the parent compound
CONSTANT Frac = 0.65 ! Unitless, fraction of parent compound metabolized to the marker residue

! Urinary elimination rate constants
CONSTANT KurineC = 0.0001 ! L/h/kg, for parent compound
CONSTANT Kurine1C = 0.14 ! L/h/kg, for the major metabolite

CONSTANT PDOSEoral = 2.5 ! mg/kg
CONSTANT PDOSEiv = 0 ! mg/kg
CONSTANT PDOSEim = 0 ! mg/kg

```
CONSTANT PDOSEsc = 0 ! mg/kg

END ! INITIAL

DYNAMIC

ALGORITHM IALG = 2
NSTEPS    NSTP = 10
MAXTERVAL MAXT = 1.0e9
MINTERVAL MINT = 1.0e-9
CINTERVAL CINT = 0.1

DERIVATIVE
! code for calulating the derivative goes here
! Cardiac output and blood flows to tissues (L/h)
QC=QCC*BW ! Cardiac output
QL=QLC*QC ! Blood flow to the liver
QK=QKC*QC ! Blood flow to the kidney
QM=QMC*QC ! Blood flow to the muscle
QF=QFC*QC ! Blood flow to the fat
Qrest = QC-QL-QK-QM-QF ! Blood flow to the rest of body

! Tissue volumes (L)
VL=VLC*BW ! Liver
VK=VKC*BW ! Kidney
VM=VMC*BW ! Muscle
VF=VFC*BW ! Fat
VLu=VLuC*BW ! Lung
VBlood=VBloodC*BW ! Blood
Vven=VBlood*0.74 ! Venous blood
Vart=VBlood*0.26 ! Arterial blood
Vrest = BW-VL-VK-VM-VF-VLu-VBlood ! Rest of body

! Dosing amounts (mg converted to umol)
DOSEoral=PDOSEoral*BW*MWmol ! umol
DOSEiv=PDOSEiv*BW*MWmol ! umol
DOSEim=PDOSEim*BW*MWmol ! umol
DOSEsc=PDOSEsc*BW*MWmol ! umol

! Multiple oral dosing using the PULSE/EXPOSURE function
CONSTANT tlen = 0.001 ! Length of exposure, oral, iv, im, or sc (h/day)
CONSTANT tinterval = 24 ! administration interval, varied dependent on the exposure paradigm
(h)
CONSTANT Dstart = 0.0 ! Initiation day of exposure (day)
CONSTANT Dstop = 5 ! Termination day of exposure (day)
CONSTANT MAXT = 1.0  ! maximum comm. interval
```

```
CONSTANT CINTC = 0.1 ! Communication interval
CINT = CINTC  ! Communication interval

Tsim=TSTOP*24 ! Tstop in hours
DS=Dstart*24 ! Initiation time point of exposure (h)
Doff=(Dstop-Dstart)*24 ! Exposure duration (h)
TimeOn=Dstart*24 ! Initiation time point of exposure (h)
TimeOff=Dstop*24+tlen ! Termination time point of exposure (h)

Exposure=PULSE(0,tinterval,tlen)*PULSE(DS,Tsim,Doff) ! Exposure paradigm
RDOSEoral=(DOSEoral/tlen)*Exposure ! Administrataion rate
RAST=RDOSEoral-Kst*AST ! Rate in the stomach
AST=Integ(RAST,0)!0.0 or Doseoral if the initial dose is twice as the subsequent dose.
RAI=Kst*AST-Ka*AI-Kfeces*AI ! Rate in the intestine
Rfeces=Kfeces*AI ! Fecal elimination rate
Afeces=Integ(Rfeces,0.0) ! Amount eliminated through feces
AI=Integ(RAI,0.0) ! Amount in the intestine
RAO=Ka*AI ! Oral absorption rate
AAO=Integ(RAO,0.0) ! Amount absorbed

! Single IV dosing to the venous
IVR=DOSEiv/timeiv
RIV=IVR*(1.0-step(timeiv)) ! Intravenous injection rate
AIV=Integ(RIV,0.0) ! Amount injected

! Single IM exposure
Rim=Kim*Aimsite ! Intramuscular absorption rate
Aim=Integ(Rim,0.0) ! Amount absorbed via IM route
Rimsite=-Kim*Aimsite ! Rate of changes in the amount of the drug in the injection site
Aimsite=Integ(Rimsite,Doseim) ! Amount of the drug remained in the injection site

! Multiple IM exposure (if needed)
!RDOSEim=(DOSEim/tlen)*Exposure
!Rimsite=RDOSEim-Kim*Aimsite
!Aimsite=Integ(Rimsite,0.0)
!Rim=Kim*Aimsite
!Aim=Integ(Rim,0.0)

! Single SC exposure
Rsc=Ksc*Ascsite ! Subcutaneous absorption rate
Asc=Integ(Rsc,0.0) ! Amount absorbed via SC route
Rscsite=-Ksc*Ascsite ! Rate of changes in the amount of the drug in the injection site
Ascsite=Integ(Rscsite,Dosesc) ! Amount of the drug remained in the injection site

! Metabolic rate
Km=KmC*BW ! h-1
```

! Urinary elimination rates
Kurine=KurineC*BW ! L/h, for the parent drug
Kurine1=Kurine1C*BW ! L/h, for the major metabolite

! ********Sub-model for the parent compound (parent drug)****************************
! Venous blood/plasma
RV=QL*CVL+QK*CVK+QM*CVM+QF*CVF+Qrest*CVrest+Riv+Rim+Rsc-QC*CV ! Rate, umol/h
AV=Integ(RV,0.0) ! Amount, umol
CV=AV/Vven ! Concentration of the total parent drug (free plus bound), umol/L
CVfree=CV*(1-PB) ! Concentration of the parent drug that is free, umol/L
CVbound=CV*PB ! Concentration of the parent drug that is bound, umol/L
CVmg=CV*MWmg ! Concentration of the total parent drug (free plus bound), unit conversion from umol/L to mg/L (ug/g)

! Arterial blood/plasma
RA=QC*CVLu-QC*CAfree ! Rate, umol/h
AA=Integ(RA,0.0) ! Amount, umol
CA=AA/Vart ! Concentration of the total parent drug (free plus bound), umol/L
CAfree=CA*(1-PB) ! Concentration of the parent drug that is free, umol/L
CAbound=CA*PB ! Concentration of the parent drug that is bound, umol/L
ABlood=AV+AA ! Amount of the total drug in the blood, umol

! Lung compartment
RALu=QC*(CV-CVLu) ! Rate, umol/h
ALu=Integ(RALu,0.0) ! Amount, umol
CLu=ALu/VLu ! Concentration of the total parent drug in the lung, umol/L
CVLu=CLu/PLu ! Concentration of the total parent drug in venous blood drained from the lung, umol/L

! Liver compartment
RL=QL*(CAfree-CVL)+RAO-Rmet ! Rate, umol/h
AL=Integ(RL,0.0) ! Amount, umol
CL=AL/VL ! Concentration of the total parent drug in the liver, umol/L
CVL=CL/PL ! Concentration of the total parent drug in the venous blood drained from the liver, umol/L
CLmg=CL*MWmg ! Concentration of the total parent drug in the liver, mg/L (ug/g)

! Metabolism of the parent compound in the liver compartment
Rmet=Km*CL*VL ! Total hepatic metabolic rate, umol/h
Rmet1=Rmet*Frac ! Hepatic metabolic rate to the major metabolite, umol/h
Rmet2=Rmet*(1-Frac) ! Hepatic metabolic rate to other minor metabolites, umol/h
Amet=Integ(Rmet,0.0) ! Amount of the parent drug that is metabolized in the liver, umol
Amet1=Integ(Rmet1,0.0) ! Amount of the major metabolite that is produced in the liver, umol

Amet2=Integ(Rmet2,0.0) ! Amount of other minor metabolites that are produced in the liver, umol

! Kidney compartment
RK=QK*(CAfree-CVK)-Rurine ! Rate, umol/h
AK=Integ(RK,0.0) ! Amount, umol
CK=AK/VK ! Concentration of the total parent drug in the kidney, umol/L
CVK=CK/PK ! Concentration of the total parent drug in the venous blood drained from the kidney, umol/L
Ckmg=Ck*MWmg ! Concentration of the total parent drug in the kidney, mg/L (ug/g)

! Urinary excretion of the parent compound
Rurine=Kurine*CVK ! Rate, umol/h
Aurine=Integ(Rurine,0.0) ! Amount, umol

! Muscle compartment
RM=QM*(CAfree-CVM) ! Rate, umol/h
AM=Integ(RM,0.0) ! Amount, umol
CM=AM/VM ! Concentration of the total parent drug in the muscle, umol/L
CVM=CM/PM ! Concentration of the total parent drug in the venous blood drained from the muscle, umol/L
CMmg=CM*MWmg ! Concentration of the total parent drug in the muscle, mg/L (ug/g)

! Fat compartment
RF=QF*(CAfree-CVF) ! Rate, umol/h
AF=Integ(RF,0.0) ! Amount, umol
CF=AF/VF ! Concentration of the total parent drug in the fat, umol/L
CVF=CF/PF ! Concentration of the total parent drug in the venous blood drained from the fat, umol/L

! Rest-of-body compartment
Rrest=Qrest*(CAfree-CVrest) ! Rate, umol/h
Arest=Integ(Rrest,0.0) ! Amount, umol
Crest=Arest/Vrest ! Concentration of the total parent drug in the rest-of-body, umol/L
CVrest=Crest/Prest ! Concentration of the total parent drug in the venous blood drained from the rest-of-body, umol/L

! Mass balance for the parent drug
Qbal=QC-QL-QK-QM-QF-Qrest ! Blood flow balance
Tmass=ABlood+AL+AK+AM+AF+Arest+ALu+Aurine+Amet ! Total amount in the body, umol
Bal=AAO+AIV+AIM+ASC-Tmass ! Mass balance, input minus output should be equal to zero at all time

! ***********Sub-model for the major metabolite (usually the marker residue)**************
! Venous blood/plasma

RV1=QL*CVL1+QK*CVK1+QM*CVM1+QF*CVF1+Qrest*CVrest1-QC*CV1 ! Rate, umol/h
AV1=Integ(RV1,0.0) ! Amount, umol
CV1=AV1/Vven ! Concentration of the major metabolite (free plus bound), umol/L
CV1free=CV1*(1-PB1) ! Concentration of the major metabolite that is free, umol/L
CV1bound=CV1*PB1 ! Concentration of the major metabolite that is bound, umol/L
CV1mg=CV1*MW1mg ! Concentration of the major metabolite (free plus bound), unit conversion from umol/L to mg/L (ug/g)
CVtotalmg=CVmg+CV1mg ! Concentration of the parent drug plus the major metabolite, mg/L (ug/g)

! Arterial blood/plasma
RA1=QC*CVLu1-QC*CA1free ! Rate, umol/h
AA1=Integ(RA1,0.0) ! Amount, umol/h
CA1=AA1/Vart ! Concentration of the major metabolite (free plus bound), umol/L
CA1free=CA1*(1-PB1) ! Concentration of the major metabolite that is free, umol/L
CA1bound=CA1*PB1 ! Concentration of the major metabolite that is bound, umol/L
ABlood1=AV1+AA1 ! Amount of the major metabolite in the blood, umol

! Lung compartment
RALu1=QC*(CV1-CVLu1) ! Rate, umol/h
ALu1=Integ(RALu1,0.0) ! Amount, umol
CLu1=ALu1/VLu ! Concentration of the major metabolite in the lung, umol/L
CVLu1=CLu1/PLu ! Concentration of the major metabolite in venous blood drained from the lung, umol/L

! Liver compartment
RL1=QL*(CA1free-CVL1)+Rmet1 ! Rate, umol/h
AL1=Integ(RL1,0.0) ! Amount, umol
CL1=AL1/VL ! Concentration of the major metabolite in the liver, umol/L
CVL1=CL1/PL1 ! Concentration of the major metabolite in venous blood drained from the liver, umol/L
CL1mg=CL1*MW1mg ! Concentration of the major metabolite in the liver, mg/L (ug/g)
CLtotalmg=CL1mg+CLmg ! Concentration of the parent drug plus the major metabolite, mg/L (ug/g)

! Kidney compartment
RK1=QK*(CA1free-CVK1)-Rurine1 ! Rate, umol/h
AK1=Integ(RK1,0.0) ! Amount, umol
CK1=AK1/VK ! Concentration of the major metabolite in the kidney, umol/L
CVK1=CK1/PK1 ! Concentration of the major metabolite in venous blood drained from the kidney, umol/L
CK1mg=CK1*MW1mg ! Concentration of the major metabolite in the kidney, mg/L (ug/g)
CKtotalmg=CK1mg+CKmg ! Concentration of the parent drug plus the major metabolite, mg/L (ug/g)

! Urinary excretion of the major metabolite

```
Rurine1=Kurine1*CVK1 ! Rate, umol/h
Aurine1=Integ(Rurine1,0.0) ! Amount, umol

! Muscle compartment
RM1=QM*(CA1free-CVM1) ! Rate, umol/h
AM1=Integ(RM1,0.0) ! Amount, umol
CM1=AM1/VM ! Concentration of the major metabolite in the muscle, umol/L
CVM1=CM1/PM1 ! Concentration of the major metabolite in venous blood drained from the muscle, umol/L
CM1mg=CM1*MW1mg ! Concentration of the major metabolite in the muscle, mg/L (ug/g)
CMtotalmg=CM1mg+CMmg ! Concentration of the parent drug plus the major metabolite, mg/L (ug/g)

! Fat compartment
RF1=QF*(CA1free-CVF1) ! Rate, umol/h
AF1=Integ(RF1,0.0) ! Amount, umol
CF1=AF1/VF ! Concentration of the major metabolite in the fat, umol/L
CVF1=CF1/PF1 ! Concentration of the major metabolite in venous blood drained from the fat, umol/L

! Rest-of-body compartment
Rrest1=Qrest*(CA1free-CVrest1) ! Rate, umol/h
Arest1=Integ(Rrest1,0.0) ! Amount, umol
Crest1=Arest1/Vrest ! Concentration of the major metabolite in the rest-of-body, umol/L
CVrest1=Crest1/Prest1 ! Concentration of the major metabolite in venous blood drained from the rest-of-body, umol/L

! Mass balance for the major metabolite
Tmass1=ABlood1+AL1+AK1+AM1+AF1+Arest1+ALu1+Aurine1
Bal1=Amet1-Tmass1 ! Input minus output should be equal to zero at all time

END ! DERIVATIVE

! Add discrete events here as needed
!  DISCRETE
!  END

! code that is executed once at each communication interval goes here
CONSTANT TSTOP = 1080
TERMT (T .GE. TSTOP, 'checked on communication interval: REACHED TSTOP')

END ! DYNAMIC
TERMINAL
! code that is executed once at the end of a simulation run goes here
END ! TERMINAL
END ! PROGRAM
```

Supplementary References

1. Foster, D. M., Jacob, M. E., Warren, C. D. & Papich, M. G. Pharmacokinetics of enrofloxacin and ceftiofur in plasma, interstitial fluid, and gastrointestinal tract of calves after subcutaneous injection, and bactericidal impacts on representative enteric bacteria. *J. Vet. Pharmacol. Ther.* **39,** 62-71 (2016).
2. Brown, S. A., Chester, S. T. & Robb, E. J. Effects of age on the pharmacokinetics of single dose ceftiofur sodium administered intramuscularly or intravenously to cattle. *J. Vet. Pharmacol. Ther.* **19,** 32-38 (1996).
3. Agriculture & Agri-Food Canada, *Ceftiofur - residues of some veterinary drugs in animal and food* (Food and Agriculture Organization of the United Nations (FAO) Corporate Document Repository: http://www.fao.org/docrep/w8338e/w8338e05.htm). (1996) Date of access: 04/02/2016.
4. Yein F. S. *et al*. Disposition and metabolism of 14C-ceftiofur sodium in swine. *International Pig Veterinary Society 11th Congress Proceedings*, 111 (1990).
5. Brown S. A. *et al*. Comparison of plasma pharmacokinetics and bioavailability of ceftiofur sodium and ceftiofur hydrochloride in pigs after a single intramuscular injection. *J. Vet. Pharmacol. Ther.* **22,** 35-40 (1999).
6. Scheer, M. Concentrations of active ingredient in the serum and in tissues after oral and parenteral administration of Baytril. *Vet. Med. Rev.* **2,** 104-118 (1987).
7. Idowu, O. R., Peggins, J. O., Cullison, R. & Bredow, J. Comparative pharmacokinetics of enrofloxacin and ciprofloxacin in lactating dairy cows and beef steers following intravenous administration of enrofloxacin. *Res. Vet. Sci.* **89,** 230-235 (2010).

8. Nielsen P. & Gyrd-Hansen, N. Bioavailability of enrofloxacin after oral administration to fed and fasted pigs. *Pharmacol. Toxicol*. **80,** 246-250 (1997).

9. Bimazubute M. *et al*. Penetration of enrofloxacin into the nasal secretions and relationship between nasal secretions and plasma enrofloxacin concentrations after intramuscular administration in healthy pigs. *J. Vet. Pharmacol. Ther*. **33,** 183-188 (2010).

10. U.S. Food and Drug Administration, *Freedom of information summary: supplemental new animal drug application, Baytril 100 injection solution, enrofloxacin, swine* (FDA NADA 141-068: http://www.fda.gov/downloads/AnimalVeterinary/Products/ApprovedAnimalDrugProducts/FOIADrugSummaries/UCM433789.pdf). (2014) Date of access: 04/02/2016.

11. Odensvik, K. & Johansson, I. M. High-performance liquid chromatography method for determination of flunixin in bovine plasma and pharmacokinetics after single and repeated doses of the drug. *Am. J. Vet. Res.* **56,** 489-495 (1995).

12. Pairis-Garcia M. D. *et al*. Pharmacokinetics of flunixin meglumine in mature swine after intravenous, intramuscular and oral administration. *BMC Vet. Res.* **9,** 165 (2013).

13. Nouws J. F. *et al*. Age and dosage dependency in the plasma disposition and the renal clearance of sulfamethazine and its N4-acetyl and hydroxy metabolites in calves and cows. *Am. J. Vet. Res*. **47,** 642-649 (1986).

14. Barnes C. J. *et al*. Comparative depletion of sulfamethazine in bob veal, fancy veal, and replacement calves. *J. Food Protect.* **53,** 154-157 (1990).

15. Duffee N. E. *et al*. Pharmacokinetics of sulfamethazine in male, female and castrated male swine. *J. Vet. Pharmacol. Ther.* **7,** 203-211 (1984).

16. Mitchell, A. D. & Paulson, G. D. Depletion kinetics of 14C-sulfamethazine [4-amino-N-(4, 6-dimethyl-2-pyrimidinyl)benzene[U-14C]sulfonamide] metabolism in swine. *Drug Metab. Dispos.* **14,** 161-165 (1986).

17. Shelver W. L. *et al*. Comparison of ELISA and LC-MS/MS for the measurement of flunixin plasma concentrations in beef cattle after intravenous and subcutaneous administration. *J. Agric. Food Chem.* **61,** 2679-2686 (2013).

18. Howard J. T. *et al*. The effect of breed and sex on sulfamethazine, enrofloxacin, fenbendazole and flunixin meglumine pharmacokinetic parameters in swine. *J. Vet. Pharmacol. Ther.* **37,** 531-541 (2014).

19. Brown S. A. *et al*. Comparison of plasma pharmacokinetics and bioequivalence of ceftiofur sodium in cattle after a single intramuscular or subcutaneous injection. *J. Vet. Pharmacol. Ther.* **23,** 273-280 (2000).

20. Beconi-Barker, M. G., Hornish, R. E., Vidmar, T. J., Dame, K. J. & Brown, S. A. Ceftiofur hydrochloride: plasma and tissue distribution in swine following intramuscular administration at various doses. *J. Vet. Pharmacol. Ther.* **19,** 192-199 (1996).

21. Schionning, S., Millerioux, L. & Hewett, G. R. Determination of tissue concentrations of desfuroylceftiofur in pigs after intramuscular injection of Excenel-TM Steril Powder. Paper presented at the European Association of Veterinary Pharmacology and Toxicology 6th Congress, p. 76-77, Edinburgh, UK, 7-11 August 1994.

22. Tantituvanont, A., Yimprasert, W., Werawatganone, P. & Nilubol, D. Pharmacokinetics of ceftiofur hydrochloride in pigs infected with porcine reproductive and respiratory syndrome virus. *J. Antimicrob. Chemother.* **63,** 369-373 (2009).

23. U.S. Food and Drug Administration, *Freedom of Information Summary: EXCENEL Sterile Suspension (ceftiofur hydrochloride)* (FDA NADA 140-890: http://www.fda.gov/downloads/AnimalVeterinary/Products/ApprovedAnimalDrugProducts/FOIADrugSummaries/ucm059120.pdf). (1996) Date of access: 04/02/2016.

24. Davis, J. L., Foster, D. M. & Papich, M. G. Pharmacokinetics and tissue distribution of enrofloxacin and its active metabolite ciprofloxacin in calves. *J. Vet. Pharmacol. Ther.* **30,** 564-571 (2007).

25. Wiuff, C., Lykkesfeldt, J., Aarestrup, F. M. & Svendsen, O. Distribution of enrofloxacin in intestinal tissue and contents of healthy pigs after oral and intramuscular administrations. *J. Vet. Pharmacol. Ther.* **25,** 335-342 (2002).

26. U.S. Food and Drug Administration, *Sulka-S ™Bolus - original approval* (FDA NADA 140-909: http://www.fda.gov/AnimalVeterinary/Products/ApprovedAnimalDrugProducts/FOIADrugSummaries/ucm054850.htm). (1989) Date of access: 04/02/2016.

27. Mitchell, A. D., Paulson, G. D. & Zaylskie, R. G. Steady state kinetics of 14C-sulfamethazine [4-amino-N-(4,6-dimethyl-2-pyrimidinyl)benzene[U-14C]sulfonamide] metabolism in swine. *Drug Metab. Dispos.* **14,** 155-160 (1986).

28. Buur, J. L., Baynes, R. E., Craigmill, A. L. & Riviere, J. E. Development of a physiologic-based pharmacokinetic model for estimating sulfamethazine concentrations in swine and application to prediction of violative residues in edible tissues. *Am. J. Vet. Res.* **66,** 1686-1693 (2005).

29. Upton, R. N. Organ weights and blood flows of sheep and pig for physiological pharmacokinetic modelling. *J. Pharmacol. Toxicol. Methods* **58,** 198-205 (2008).

30. Leavens T. L. *et al*. Development of a physiologically based pharmacokinetic model for flunixin in cattle (Bos taurus). *Food Addit. Contam. Part A Chem. Anal. Control Expo. Risk Assess.* **31,** 1506-1521 (2014).

31. Achenbach, T. E. Physiological and classical pharmacokinetic models of oxytetracycline in cattle. Thesis, Simon Fraser University, British Columbia, Canada (2000).

32. Rantala M. *et al*. Efficacy and pharmacokinetics of enrofloxacin and flunixin meglumine for treatment of cows with experimentally induced Escherichia coli mastitis. *J. Vet. Pharmacol. Ther.* **25,** 251-258 (2002).

33. Messenger, K. M., Papich, M. G. & Blikslager, A. T. Distribution of enrofloxacin and its active metabolite, using an in vivo ultrafiltration sampling technique after the injection of enrofloxacin to pigs. *J. Vet. Pharmacol. Ther.* **35,** 452-459 (2012).

34. Yuan, Z. H., Miao, X. Q. & Yin, Y. H. Pharmacokinetics of ampicillin and sulfadimidine in pigs infected experimentally with Streptococcus suum. *J. Vet. Pharmacol. Ther.* **20,** 318-322 (1997).
